# Medico-Chirurgical Transactions. Vol. XIX. 1835

**Published:** 1836-01-01

**Authors:** 


### MEDICO-CHIRURGICAL TRANSACTIONS; published by the ROYAL MEDICAL AND CHIRURGICAL SOCIETY OF LONDON. Volume the Nineteenth. Oct. 1835.

THE eighteenth volume was not complete by one-half; but, in consequence of the royal charter accorded to the Society, they have deemed it best to affix an index to the half-volume, and commence with the nineteenth volume at once. The charter and bye-laws are prefixed; and now the "ROYAL MEDICAL AND CHIRURGICAL SOCIETY" is fairly launched into the world. We wish it success.

The volume before us contains nineteen articles, a large proportion of which are of a pathological character. As they are not connected by any indissoluble, or even natural ties, we shall select the following for notice in the present Number of this Journal, and complete our notice of the remainder in our next. Some of the shorter papers will be found by our readers in our Periscope.

The contents of the volume may be arranged under three, or perhaps four heads:—Medical, surgical, and physiological. Pathology can hardly be separated from any of the above branches. We shall take the medical department first.

### I. CASE OF PULMONARY PHLEBITIS. By ROBERT LEE, M.D. F.R.S.

A young woman, 20 years of age, was delivered of her first child in the Parochial Infirmary of St. Martin's, 30th Nov. 1833. The labour was natural, and she went on well for five days, when a rigor occurred, followed by a profuse perspiration, dyspnœa, pain in the left side of the chest, and over the abdomen. The pulse was rapid. Bleeding, calomel and opium, and purgatives were employed. Diarrhœa and deep-seated pain in the region of the uterus were added to the other symptoms. She died on the 29th Dec. the pulse varying from 120 to 150, two shivering fits occurring each day, followed by profuse perspiration. There was some cough, with mucous expectoration. Dr. Lee got permission, through the assistance of Mr. Gosna, to examine the body. The following is the report.

"*Inspection.* The uterus had undergone the reduction of volume usually observed a month after delivery, and no morbid appearance was at first discernible in any of the abdominal or pelvic viscera. On slightly drawing up the uterus towards the brim of the pelvis, the fimbriated extremity of the left fallopian tube was seen adhering by lymph to the back part of the body and cervix of the uterus, and in the folds of the broad ligament near the left ovarium, an abscess about the size of a walnut was observed. On the left side, the veins of the cervix and body of the uterus were all gorged with purulent fluid, and lined with thin, ash-grey coloured false membranes; in the muscular tissue of the anterior part of the cervix uteri, was a small sloughy abscess. The left obturator, and the uterine branches of the internal iliac vein, were filled up with a soft yellowish coagulum of lymph, which adhered to the inner coat of the vessel, and contained pus in its centre, and was continued downward along the external iliac, terminating abruptly a little above Poupart's ligament. It also extended upward about half an inch into the common iliac, and terminated in a loose blunt point. A firm clot of dark-coloured blood, which did not adhere to the lining membrane, filled the greater part of the remaining portion of the common iliac vein. Near the termination of this vessel, a coagulum of lymph, similar to that in the external and internal iliac veins, existed, which closely adhered to the inner membrane, and passed into the vena cava.

The whole vena cava, between its commencement in the common iliac and the entrance of the hepatic veins, was lined with a soft, yellowish-coloured pultaceous mass of lymph and pus, which adhered, in some parts loosely, and in others firmly, to the internal tunic of the vessel. The entrance of the right spermatic vein into the vena cava was closed with a firm false membrane, and the coats of the vena cava, above and below, were twice their natural thickness. The greater portion of the right spermatic vein was lined with dense false membranes of a bluish colour, and its coats were thickened and contracted.

The serous surface of the middle and inferior lobes of the lungs on the right side was covered with soft, yellow lymph, and their substance was hepatized. On cutting into the left inferior lobe the pulmonary texture was found dense, and of a dark red colour; and pus escaped from two branches of the vein, which traversed this portion of the substance of the lung. On careful inspection of the venous trunk and branches of the left inferior lobe of the lungs, all the appearances usually seen in inflammation of veins in other internal organs of the body were observed. The trunk of the vein, near its entrance into the left auricle of the heart, was found plugged up with a soft, yellowish-coloured clot of lymph, firmly adherent to the inner surface of the vessel, and extending into several of its principal branches in the substance of the lung. The coagulum of lymph on the outer surface was of a bright scarlet colour, when separated from the vein. The smaller branches of the vein, into which the solid lymph did not extend, were filled with pus, and in some parts were coated with a delicate layer of yellow lymph." 47.

The preceding is the only observation of pulmonary phlebitis which has fallen under Dr. Lee's notice. The case is remarkable for the great extent of the disease in other veins of the body. There can be little doubt that we have too much neglected an examination of the interior of vessels.

### II. Singular Laceration of the Peritoneal Coat of the Uterus. By W. H. Partridge, Esq.

Mrs. B. was suddenly seized, when in the eighth month of pregnancy, with abdominal pains and bilious vomiting. In two hours serous, and then sanguineous discharge per vaginam. Opium, and afterwards some brandy, allayed the vomiting; but labour came on in six or seven hours from the commencement of the attack, and she was delivered of twins in two hours more. Great exhaustion succeeded—and the abdominal pain continued. An opiate was administered, without relief. She expired shortly afterwards.

On opening the abdomen, a quantity of thin, dark-coloured blood was found, amounting to about 40 ounces, but no coagula. The uterus was well contracted, and, on its posterior surface, were several transverse lacerations, all more or less curved in form, with the convex part towards the fundus uteri. They averaged from half an inch to two inches in length, and were very various in depth. The cause of these curious lacerations appears to us inexplicable. There are a few similar cases on record; but they lead to no practical results.

### III. OBSERVATIONS ON FIBRO-CALCAREOUS TUMORS IN THE UTERUS. By Dr. R. LEE.

Perhaps we should have carried our division farther, and left a head for midwifery—we beg pardon, OBSTETRICY. The *pure* physician and surgeon are fast losing some valuable portions of practice—the physician-accoucheur hemming them in on one side, and the general practitioner driving them out of the other! Then they are clipped down by the dentists, the oculists, the aurists, the spine-doctors, the liver-doctors, the lung-doctors, the gout-curers, the shampooers, the vapour-doctors, the pox-doctors, *cum multis aliis.* However, we do not quarrel with these divisions of labour, seeing that they are productive of good in other sciences, and are probably beneficial in the healing art.

In the fifteenth and sixteenth volume of these Transactions, Dr. Lee endeavoured to give some account of those uterine affections which are the products of inflammation in one or more textures which enter into the composition of the organ: in the present volume he takes up the subject of tumors, not malignant;—and especially the fibro-calcareous tumors and polypi—occasioning some of the most important affections of that class of uterine diseases. We regret that we shall not be able to do sufficient justice to this important paper, evincing great research, as well as accurate observation. We shall do our best, however, to comprehend its principal features in this article.

The fibrous tumor (fleshy tubercle of W. Hunter) is sometimes met with as small as a pea—in other cases, as large as a cricket-ball, or larger. It is generally of a globular form, or kidney-shaped, presenting, when cut into, a laminated or radiated semi-cartilaginous structure. In some cases the structure is granular, appearing like a congeries of smaller tumors, each having a thin capsule. The centres are sometimes found softened or liquid. In other cases, the density seems to increase with age, till the whole mass becomes cartilaginous, without vessels containing red blood. Or calcareous depositions are gradually formed in the substance of the tumor, till it is partially or wholly converted into carbonate and phosphate of lime. They vary in density, from that of pumice-stone to marble. In many cases which are glanced at, from other authors and contemporary practitioners, these tumors were accompanied by little or no pain. In the following case there was malignant ulceration in the uterus, and the sufferings were protracted.

*Case.* Mrs. B. aged 62, had suffered for many years from weight and uneasiness in the back, loins, and hypogastrium, with purulent or sanguineous discharge. She was married, but had no children. She ceased to menstruate at the age of 45 years. On examination, the hollow of the sacrum was found occupied by a large tumor, hard and connected with the posterior part of the uterus. The os uteri had undergone little change; but the fætor of the discharge and constitutional symptoms led to the suspicion of organic disease. In the course of severe suffering, some small irregular concretions escaped from the uterus, with temporary relief—and this occurred several times afterwards. In the month of November, 1833, after a long day's journey, she was attacked with rigor, vomiting, acute pain and other symptoms of peritonitis, which carried her off in 48 hours.

*Inspection.* Dr. Webster was present at the examination. The usual effects of peritonitis were present. The fundus and body of the uterus were destroyed by malignant ulceration; and to the posterior part of the organ was attached a large fibro-calcareous tumor, filling up the hollow of the sacrum, and displacing the rectum. The ulceration had extended through the parietes of the uterus to the tumor. The concretions were chiefly composed of carbonate of lime and animal matter.

A somewhat similar case has been recorded by M. Louis. And several others have been collected by our indefatigable author, for which we refer to the volume itself.

Fibrous tumors, when situated between the peritoneum and muscular coat of the uterus, give rise to no irritation, hæmorrhage, or derangement either of uterine function or general health. But when they attain a large size, and occupy a considerable space of the abdominal cavity, they produce all the numerous consequences of enlarged ovaria, from which, indeed, during life, they can seldom be distinguished. Under the peritoneum of the uterus, they do not prevent pregnancy; but when adherent to the posterior part of the body or neck of the uterus, they sometimes produce fatal consequences both to mother and child, by impeding its progress through the pelvis.

"When fibrous tumours are imbedded in the proper tissue of the uterus, women are frequently barren, or if they become pregnant, abortion takes place in consequence of the uterus being incapable of undergoing the necessary developement in the latter months of gestation. When the ovum is not prematurely expelled, death may take place in such cases from uterine hemorrhage, soon after delivery. M. Chaussier saw a woman die from flooding, soon after giving biith to a full grown child, and there was a large fibrous tumour in the posterior walls of the uterus. This tumour was not situated so as to present an obstacle to the passage of the child through the pelvis, but soon after delivery it was perceived that the uterus had not the power of contraction. Profuse hemorrhage took place from that part of the uterus in which the tumour was lodged, the flow of blood could not be arrested, and the patient died." 111.

Dr. Lee examined the body of a woman who died after embryotomy of a still-born hydrocephalic child. The liquor amnii amounted to sixteen pints. Profuse uterine hæmorrhage followed the placental delivery—she died on the third day. He found a hard fibrous tumor, the size of a hen's egg, embedded in the muscular coat of the uterus, where the placenta had adhered. But we are unable to follow the talented and unwearied pathologist through this long and important paper. We can only make room for one short extract in conclusion.

"With respect to the treatment of the various tumours which have now been described, I have few observations to offer. Iodine, mercury, and all other remedies have little effect either in arresting their growth or promoting their absorption. Women who have fibrous tumours formed in the walls of the uterus, should avoid mechanical pressure of the hypogastrium, violent bodily exertion, and every other cause which may excite inflammation or a determination of blood to the organs within the pelvis. Where congestion has taken place, it should be removed by local blood-letting, mild cathartics, and anodynes. Profuse uterine hemorrhage should be controlled by rest in the recumbent posture, cold application to the hypogastrium, and the internal use of the acetate of lead.

When any of these tumours pass through the os uteri into the vagina, they may be removed by the ligature or by the knife. If the root is soft and slender, the tumour may easily be twisted off by the forceps. In the course of the last twenty years, Dupuytren states, that he has removed two hundred uterine polypi by excision. Hemorrhage has only occurred twice in all the cases, and in both instances it was permanently arrested by the tampon. In eight or ten cases, after the application of the ligature, death took place from the absorption of pus into the system.

Where the root of a tumour is large and vascular, I am of opinion that a ligature should previously be passed around it, at as great a distance from the os uteri as is compatible with the removal of the disease." 132.

This paper is a highly valuable one, and deserves attention. As the talented author is now lecturer on the practice of midwifery in the school of St. George's Hospital, the pupils of that school will enjoy the advantage of receiving the lessons of one of the most philosophical professors of midwifery in this country.

IV. CASES OF WARTY TUMORS IN CICATRICES. By CÆSAR HAWKINS, Esq. Surgeon to St. George's Hospital, and Lecturer on Surgery in the Hospital School.

This is the second paper of the volume. Mr. Hawkins very properly points out the confusion that arises from the vague signification attached by various persons to the term malignant. A morbid growth or morbid alteration of structure may be incurable, that is, may be incapable of those actions that lead to its removal. Another morbid growth may not only be incurable, but may contaminate contiguous or distant parts through the medium of absorption or the absorbents. It is clear that the two conditions are widely separated, and ought to be so. But in practice, the same term, malignant, is too frequently applied with the effect of confounding or destroying the distinction.

But morbid changes of structure are not simply incurable without contaminating neighbouring parts, and malignant by contaminating such parts and the general system—they sometimes possess the power of contaminating contiguous structures, but *not* of poisoning the system at large. As Mr. Hawkins justly observes—

"We want some word for those diseases which *do form a new structure* capable, apparently, of contaminating the surrounding parts, so that the removal of the whole of the altered structure is necessary, but which *do not*, as far as I know, produce any contaminating influence upon the absorbent glands, and have no tendency whatever to reappear in a distant and unconnected part of the body. Such a disease is familiar to most surgeons in the skin of the face of elderly persons, and is often, but I think erroneously, called *cancerous* and *malignant*, since if the new structure at its basis be completely taken away, there need be no apprehension of any return of the disease, either in the same part or elsewhere: or at least if the new structure really possesses the nature of *cancer*, it must be clearly understood that the disease is *cancerous* and *malignant* in the very owest degree. Of this kind also is the disease which I purpose to describe by he recital of a few cases which have fallen under my observation, and which, s far as I know, is not described in any surgical writings." 21.

If we recollect rightly, Sir B. Brodie published a case of the disease Mr. H. is now describing in the London Medical and Physical Journal. It was facetiously styled in some of the medical journals of that time, "a case of cat-o'-nine-tails tumor." Let us see what the characters of this disease are.

"The tumour," says our author, "which I will call the WARTY TUMOUR OF CICATRICES, makes its appearance in some old scar, many years after the injury which has produced it has been healed, whether a burn, a cut, or a laceration of the skin; and it arises equally from a flogging or a scald, in which the skin alone has been injured, or from a cut or gun-shot wound, which injures also the tendons or bones below the skin, and makes a more complicated cicatrix. There appears in the first place a little wart, or warty tumour, in the cicatrix, which is dry and covered with a thin cuticle, but which soon becomes moist, and partially ulcerated, like the warts of mucous membranes, from which a thin and offensive, and semi-purulent fluid is secreted. In this stage it gives no pain nor inconvenience." 21.

*Case* 1. This illustrates the stage above described. A man had been repeatedly flogged in India, and in the cicatrix left by the lashes several warts sprang up, which coalesced to form a tumor; the probe passed between the warts to the basis of the disease. Around the tumor the skin was of a dark and livid colour, and studded with several smaller warts. Sir B. C. Brodie removed the tumor, then about the size of a small apple, eleven years after the infliction of the last flogging, and no return of the disease ensued.

In the second stage of the disease, proceeds Mr. Hawkins, the growth of the tumor becomes more rapid, the warty appearance being in some measure lost, a more solid substance projecting from the diseased skin, which bears much resemblance to the fungus of fungus hæmatodes; the formation of fresh warts being still seen around the tumor, and preceding the change which has been alluded to. The tumor is very vascular, and bleeds when touched, but its irregular surface still allows the probe to pass through its structure, except where it is most firm.

*Case* 2. A man, æt. 45, was admitted into St. George's Hospital, April 18th, 1827, under the care of Mr. Jeffreys.

There was a large tumor connected with the skin of the back, somewhat elevated, and with the edges overlapping the surrounding skin, which was drawn in and puckered round the tumor. The tumor was about five inches in diameter, and the skin appeared to be partly connected with the spinous processes of the dorsal vertebræ, and with the spine of the scapula. The tumor was warty and irregular, and had an ulcerated surface, discharging a thin, sanious manner. The man's countenance was sallow, the appetite however not impaired, the bowels in general constipated, and his sleep at night disturbed by shooting pain in the back.

Twenty-seven years previously to his admission this man had been flogged, and had suffered for eighteen months from the effects of that punishment. The cicatrix, however, remained quite well, until six months prior to his admission, when a piece of wood falling on it and grazing it, a small lump soon afterwards appeared in the part, and ulcerated. The tumor progressively increased till it attained the dimensions and the character which have been described.

The tumor was removed nine days after his admission by an operation, four days after which he died of phlebitis, that would seem to have commenced before the operation was performed.

*Case* 3. Susan Farrington, æt. 28, admitted into St. George's Hospital Oct. 23d, 1833, under the care of Mr. Hawkins.

The left leg and foot had been severely scalded when she was a child. The sore was more than a year in healing, and the surface of the cicatrix had, since then, frequently ulcerated. The last recurrence of ulceration had been four months prior to her admission, and two months and a half before that time, the sore had displayed the characters we shall describe.

"There was a prominent tumour, about two inches and a half above the surrounding skin, which was four or five inches long, and extended more than two-thirds round the leg. The surface had the usual irregular warty appearance of these tumours, and discharged a very fetid pus. The cicatrization of the former scald extended from the toes to very near the knee, and was wrinkled towards the tumour. The tumour allowed the probe to pass through it very readily, and when thus examined in various directions, there seemed to be no softening of the periosteum of the tibia, nor of the fascia of the leg.

She had latterly become thin and out of health, with a furred tongue, and quick and weak pulse, and more or less restlessness, from excessive pain and irritation in the tumour." 26.

The patient at first improved a little under the judicious treatment of Mr. Hawkins. But then the tumor rapidly extended, and amputation was proposed, the extent of ulcerated surface rendering excision of the tumor impossible. The patient would not at first consent, but she subsequently altered her determination, and the limb was removed below the knee. The stump healed, and no return of the disease has taken place.

"It will be seen (says Mr. Hawkins), on examination of the preparations, that the tibia was perfectly healthy, excepting an addition of new bone from common inflammation, and that the disease had not extended through the fascia to which it adhered. The drawing was taken about three weeks before the operation, when the tumour began to lose some of its distinctive warty appearance, by becoming somewhat sloughy on the surface, and by the warts becoming more solid and smoother in their prominent extremities, so as to resemble fungus hæmatodes, or the fungous kind of cancerous tumours.

I allude especially to the perfectly sound condition of the tibia, because I believe many gentlemen, who saw the leg, were of opinion the bone must have been diseased, and must have given origin to the tumour. I have placed, however, upon the table the cast of a case of a prominent fungus of a different kind, which is occasionally formed over carious bone, and which ought to be carefully distinguished from the tumour which I am endeavouring to describe. This fungus grows to the same height as the warty tumour, and resembles it in some measure in appearance, but even in the cast perhaps the difference may be perceived between them. In this exuberant growth from the cancellous structure of a bone, the projections are more like granulations; they are softer and redder than the warty tumour, and none of the peculiar changes in the skin around can be detected, which, I believe, uniformly precede the growth in question; on the contrary, the circumference of the diseased parts has the appearance of a common ulcer of the skin, and if the carious part of the bone on which the prominence depends be carefully dissected out by the chisel, the ulcer in the skin will readily heal.

But while I wish to assert the origin of the warty tumour from the texture of the skin, I am perfectly aware that a disease of some bone may be *added* to the alteration of the skin, as was shewn in the last case, or as the following case may also prove." 29.

*Case* 4. John Colley, æt. 45, admitted into St. George's Hospital, July 10th, 1833, under the care of Mr. Babington.

Twelve years ago, he cut the heel through the tendo Achillis so deeply, as to expose the bone. The wound healed in three months, but the ankle and instep continued stiff. Two years afterwards an ulcer formed in the cicatrix, and never subsequently cicatrized completely.

On his admission, a warty ulcer existed around the heel, at the bottom of which exposed bone could be felt. The tibia was enlarged, and the joint stiff. The leg was amputated, and the patient did well. On examining the ankle, the bone of the heel was found inflamed, and rough and scabrous, as in common inflammations, but without any appearance of the disease having caused any other alteration of its structure.

It appears then that the tumour may be easily and safely removed from any part of the body. In the leg, indeed, the removal of the whole limb must generally be preferable where the tumour is at all extensive ; but if its size admits of excision, there need be no fear of the disease being re-formed in any texture except the skin. Still, however, if there be any doubt whether the bone below may have become carious, or otherwise diseased, from the proximity of the tumour, a portion of it may be taken away without adding to the length of confinement." 30.

*Case* 5. John Callcott, æt. 49, admitted into St. George's Hospital May 28th, 1828, under the care of Sir Benjamin Brodie.

He had a yellow, wart-like fungus, about the size of a crown-piece, which rose above the skin, and through which some bone was felt ; this was situated in the centre of some old cicatrices. He had received a blow on the shin from an anchor, twenty-seven years previous to his admission, which was followed by a large abscess, out of which some dead bone had been taken while in a naval hospital, after which the wound healed. Fourteen months ago he received another injury, which was also succeeded by an abscess, at the bottom of which the bone was exposed. The exposed bone was believed to be dead, but as it was not loose, he left the hospital till it was in a fit state to be removed ; soon after this the fungus formed, and he was re-admitted, when the tumour seemed to be connected with the bone or the periosteum, or both. June 5th, the tumour being removed with the periosteum, to which it was fixed, a portion of the bone which seemed to be more vascular than usual was taken away with the trephine, so as to expose the medullary canal. The vascularity did not extend more than a quarter of an inch in depth, and the bone was not otherwise altered. The wound healed well, and the man has since continued free from disease.

Mr. Hawkins observes, what must, indeed, be obvious, that the knife is much preferable to caustics, as a means of removing these morbid productions. The knife is at once the less irritating, the more expeditious, and more certain of the two. We have hitherto seen the ulcer arrested in its progress to its termination, by the aid of surgery. To complete the picture, it is necessary to study it unchecked by art—to observe its ravages, and the mode in which it destroys the patient.

"After," says our author, "the tumour has become solid and prominent, a new action takes place in it, and the tumour ulcerates and sloughs alternately, with a great deal of pain and suffering, and it is destroyed down to its basis, so as to present the appearance of a foul excavated ulcer, except in its circumference, where the skin is raised, thickened, and everted, and from time to time warts are generated, which again ulcerate and slough, till the patient becomes gradually worn out by suffering, but without having at all the sallow and peculiar aspect of a person dying of a malignant disease ; and on examination of the body, no disease of the absorbent glands is found, nor is there, as far as I know, any sign of malignant disease in the interior of the body. This termination of the disease I witnessed in the following case." 23.

*Case* 6. James Sturgess, admitted under the care of Mr. Hawkins, Jan. 18th, 1832.

Sixteen years previously, he had a severe burn of the back, the cicatrix of which extends from the sacrum to the scapulæ. The sore healed, and the cicatrix continued sound till eighteen months before his admission, when "a small pimple" made its appearance. He picked it off; an ulcer formed, and it progressively extended. On his admission, the tumor which previously existed had disappeared, and an excavated ulcer, eight inches by six in diameter, was left in the loins.

No treatment availed to check the disease, which was too extensive for excision. In July, the patient died exhausted. The disease, even then, had in no part destroyed the fascia of the back, and, except in one or two places, where the sloughing had been most severe, the cutaneous basis still remained, although very thin.

"I shall be glad," concludes our author, "if the remarks I have thus presented to the Society, enable any surgeon to recognize this disease in its early stage, when it may be removed by the knife, without wasting time in the use of remedies, which seem to exert no substantial influence over its growth, and, if unsuccessful, will do much harm. The excision of the tumour, or warty ulcer, may thus prevent the necessity for the amputation of a useful limb, as in several of the cases I have related, or prevent the patient from being worn out by a disease that might have been eradicated, while its size still allowed of the operation.

If, again, the surgeon has dissected out such a tumour, or has removed a limb, when the tumour was too large to admit of separation, still it will be a great point to quiet his own and his patient's anxiety, by a confident assurance that the disease is not in the least malignant, as cancer is malignant, but is, on the contrary, entirely local in its origin, and does not contaminate even the adjacent parts, except in a very trifling degree, so that no future mischief need be apprehended." 34.

On the whole, this must be regarded as an useful and interesting contribution to surgical pathology.

### V. CASES OF FRACTURE OF THE NECK OF THE FEMUR, WITH THE APPEARANCES OBSERVED AFTER DEATH. By J. HOWSHIP, Esq. &c.

These cases are possessed of interest, because they are attested facts that bear on the vexata questio—whether fractured neck of the thigh-bone, internal to the capsule, does not admit of ossific union.

There are two ways of putting this important question—first, it may be asked if bony union *ever* takes place, or, secondly, it may be inquired if the union is not, in the *majority*, the vast majority of cases, ligamentous? He must be a hardy disputant who would affirm, that bony union never does and never can occur. A life, a century, all past experience and all present, were the observation perfectly exact and the record strictly true, would be obviously insufficient to prove such a position; for Nature's combinations are infinitely varied, and, even if such a thing had never been, we could not affirm that it never would be. Independently of this consideration, facts have been brought forward in relation to the present subject, which would seem to prove that bony union of fractured cervix femoris, within the capsule, has actually occurred.

But, although experience is insufficient to determine the impossibility of bony union, it is perfectly competent to pronounce if it generally occurs or not. So far as facts have gone, they shew that, under ordinary circumstances, the union is by ligament. Yet facts are still wanting to give to this law the precision and completeness of which it is probably susceptible. Mr. Howship has detailed a series of cases, in which death occurred at various periods after the reception of the injury, and in which dissection exhibited, in each instance, the actual condition of the parts at those epochs. Such cases are obviously possessed of much practical interest, if not importance. We shall present an outline of them.

Case 1.—*Fractured Cervix Femoris—Appearances at three Weeks.*

The patient was 76 years of age. On the day after the accident, the limb was an inch and a half shorter than its fellow, and there was eversion of the foot. Three weeks after the fall she died.

"Dissection, *April 25th*, 1829. I found the fracture oblique; above, it passed through the cartilaginous margin of the head of the bone, extending obliquely round, and separating the lower part of the neck three-fourths of an inch below its junction with the head.

The separation of the bone was complete, but the ligamentous investment covering the cervix posteriorly was entire; at the lower portion of the anterior surface a single fibre of this texture remained, restricting the edges of the fracture to the distance of half an inch. The neck of the femur had lost half an inch of its length by absorption.

The synovial fluid was deeply tinged with blood, and between the fractured surfaces small masses of coagulated blood were observed, together with a minute detached cancellated fragment of bone. The cartilaginous surfaces of the joint were much discoloured, and appeared as if their texture was ecchymosed; these surfaces, soiled with grumous blood, it was necessary to wipe clean, when it was manifest the cartilage itself was stained of a dull red hue, from blood diffused apparently through its substance. There was no appearance of any attempt towards union.

The preparation demonstrates the capsule much thickened and ecchymosed; and the round ligament of a livid red colour, but so wasted as to be scarcely perceptible." 3.

Case 2.—*Fractured Cervix Femoris—Appearances at two Months.*

C. D. æt. 75. At first, after the accident, there was no crepitus, nor was there any shortening of the limb, which was only slightly everted. In about six weeks, the limb was found to have gradually shortened about one inch.

"Dissection, *June 19th*, 1831. I found the capsule of the right hip much thickened and condensed, especially above the articulation. The fracture passed round the neck of the femur near its head. The posterior half of the ligamentous expansion covering the neck of the bone remained entire, with the exception of two or three small openings. Indeed, the degree of thickening and compactness of texture, in this expansion, forms one of the most interesting characters in this specimen. Inferiorly, the fracture left the margin of the upper fragment sharp; and the trochanters drawn upwards, as interstitial absorption advanced, the inferior portion of the ligamentous investment had ulcerated; a similar change having exposed what remained of the inferior part of the neck of the femur.

Between the fractured portions, were several light, tender, fibrinous bands, tinged with blood, connecting the cancellous surfaces to each other. The neck of the bone had lost three-fourths of an inch of its length by absorption. The round ligament entire, was discoloured by blood effused into its interstitial texture; a similar tinge having extended itself into the fatty substance surrounding the attachment of the ligamentum teres at the bottom of the acetabulum; appearances still visible in the preparation." 4.

CASE 3.—*Fractured Cervix Femoris—Appearances at Five Months.*

E. M. æt. 78. There were immediate shortening to the extent of an inch, and slight eversion of the foot, but no crepitus.

DISSECTION, *Dec. 5th*, 1828. "I found the fractured head of the femur separated within the capsule, which was thickened, compact, and ecchymosed. The head of the femur, raised from its place, enabled me to perceive that the cartilaginous surfaces were of a dark red colour, but not ulcerated; the round ligament ecchymosed, wasted, and pulpy. The soft matter occupying the cancellous fractured surface of the head, was a grumous granular mass. What yet remained of the fractured neck was undergoing absorption; the surface perforated with numerous foramina; filled with a soft granulated substance, similar to that just mentioned. At one point, the lower margin of the fractured neck was attached, by a fibrinous band, to the corresponding part of the head of the bone, and also to the capsule of the joint.

From the exposed cancellated surface of the cervix femoris, in this specimen, a flat cartilaginous plate is seen standing out in a vertical position; an appearance which, as the cartilages of the joint are still entire, it is difficult to explain." 6.

CASE 4. *Fractured Cervix Femoris—Ligamentous Union at Five Months.*

M. C. æt. 66. After the accident the limb was shortened two inches and a half; the foot everted.

DISSECTION, *Nov. 22d*, 1829. "In the hip-joint the neck of the femur appeared very much shortened, but not disunited from the head, which remained perfect, and covered with a thickened capsule. The capsule opened; the head of the bone was gently raised from the acetabulum. The round ligament was so wasted as to have become entirely disunited, at its insertion upon the femur. Some shreds of its substance still remained attached at the bottom of the acetabulum, and are still visible in the preparation; but in the depression upon the head of the femur, no trace remains. It appears that when fractured the head of the femur had been separated high up, and the neck of the bone subsequently absorbed. The head of the femur was no longer separated; for the neck removed by absorption, and the parts steadily retained in apposition, the head of the bone had settled itself so firmly into the space between the trochanters, that it appeared, at first sight, a specimen of ossific union.

To ascertain the precise state of the uniting medium, I divided the head and shaft longitudinally with a saw, in which operation the attachment of the parts suffered no apparent disturbance.

The section of this specimen in the preparation, presents a perfect example of compact ligamentous union. The fractured surface of the head, and basis of the neck, are seen so closely in contact as to exhibit only an interposed lamina, scarcely thicker than pasteboard, of compact ligamentous substance." 17.

Case 5. *Fractured Cervix Femoris—Appearances at Ten Months.*

L. M. æt. 79. She fractured the neck of the right humerus and right femur. There were shortening and eversion of the lower extremity.

Dissection, *Oct.* 13*th*, 1832. "I found the uniting medium of the fractuced humerus so soft as to admit elastic motion.

The capsule of the hip thickened; the fracture had separated the neck irregularly, about half an inch below its junction with the head. There were no fibrous bands to connect the fractured parts together; but the surface of the upper fragment was fairly covered with cartilage, except a small space at its lower part, the size of a split pea, presenting a hard, polished, ivory-like substance.

The ligamentous investment of the upper and back parts of the neck was entire, attaching the head and basis of the neck to each other. The neck of the femur was nearly absorbed; the part remaining being modelled into a little crutch, exactly fitted to receive and support the head, and almost entirely covered with cartilage. At the upper margin of the neck, and beneath the ligamentous investment, was a small flattened piece of cancellated bone, covered with cartilage, attached to the ligament, yet entering into the moving fabric of the new joint.

The round ligament, pulpy, and discoloured by ecchymosis, (although no fluid tinged with blood was found in the joint,) was attached by both its extremities; yet the cartilages and bone were ulcerating deeply away, by the agency of red fleshy granulations, still visible in the preparation; springing from within the bone surrounding each insertion of the ligament." 8.

Case 6. *Fractured Cervix Femoris—Appearances at Twenty-two Months.*

E. B. æt. 79. On the same day, there was slight eversion, but no shortening of the limb. Afterwards shortening gradually occurred.

Dissection, *May* 1830. "The limbs laid straight; the right leg and foot, much everted, proved to be three inches shorter than the left. Exposing the capsule, and opening the joint, I found the head of the femur separated from the shaft, by a fracture passing round near the margin of the head. The cancellated surface of the head was at many points covered with minute nodules of fine cartilage.

The neck of the femur was gone; part of the space between the trochanters being covered with a ligamentous fibrine, the intervals presenting open cancelli, with minute nodules of cartilage, similar to those on the opposite surface of the head. From the upper part of the basis of the neck of the femur, many rounded projections of bone, covered with cartilage, were put forth, over which the thickened capsule moved with facility.

A strong fibrinous band, three-eighths of an inch long, connected the upper margin of the head to the opposite surface of the neck. A second strong band connected the neck of the bone to the capsule, on the upper edge of the acetabulum.

The capsular ligament, on its superior part, was half an inch thick, where its breadth was so diminished as to have brought the margin of the trochanter nearly into contact with the acetabulum. Fibrinous effusion, not confined to the seat of fracture, presented itself on most parts of the synovial membrane: one little mass, attached by a narrow basis to the anterior part of the capsule, was nearly the size of a scarlet bean; another, entirely detached, in a recess within the joint, had assumed all the characters of a loose cartilage, a fine elastic structure, and smooth surface.

The round ligament, although visible in the preparation, is exceedingly wasted and shrunk." 10.

Case 7. *Fractured Cervix Femoris—Partial Ligamentous Union at Eight Years.*

R. S. æt. 70. The accident was immediately succeeded by some shortening, which afterwards gradually increased. At the end of three years, the shortening amounted to two inches, and there was trifling eversion of the limb. In May, 1833, eight years after the accident, she died, having been enabled to walk well with a stick before her death.

*Dissection.* The fractured surfaces were connected by an unusually strong ligamentous adhesion, to demonstrate which required the cutting away of nearly the whole capsule. The fracture had followed the line of the margin of the head of the femur, the neck being nearly absorbed. Anteriorly, a line or web of strong, short, fibrinous bands is seen, connecting the head of the femur, and upper part of the acetabulum, to the basis of the neck; these attachments extending from the upper to the lower margin of the acetabulum. Posteriorly, the fractured surfaces are seen attached by one thin band of fibrine only.

Inferiorly, the capsule, at one point, may be observed to be united by adhesion to the margin of the head of the bone.

Case 8. *Fractured Cervix Femoris—Appearances at Fourteen Years.*

S. M. æt. 50, fell on the hip, and both limbs being of the same length, she was pronounced, in an infirmary, to be labouring only under a bruise. In three months she walked well without a stick, and was afterwards fit for every laborious occupation. Twelve years after the accident the left leg was two inches shorter than the other, and there was slight eversion of the limb.

Dissection, 14 *years after the accident.* The line of fracture was about an inch from the margin of the head. The part of the neck remaining between the trochanters was so nearly absorbed as to have left only a gentle elevation towards the middle of the space, answering to a depression on the opposite surface. Both surfaces were covered with a copious secretion of cartilage, on some parts deposited in flattened nodules, on others presenting an even surface.

The capsule was condensed as usual. In the substance of the capsule, above, a large bursa had formed, between the margin of the trochanter and edge of the acetabulum. There was a free effusion of fibrine into the joint; and the head of the bone was so closely adherent to the capsule, as to prevent its being raised from the acetabulum, to ascertain the state, or indeed, the existence, of the round ligament.

There were no fibrinous bands connecting the fractured surfaces; the only attachments of that kind were those just mentioned, the apparent object of which was, to steady the head and neck of the bone, in a particular position, and give freedom and facility of motion to the new joint.

It is evident, in this case, that when the weight of the body, in walking, was placed on the limb, the action between the femur and pelvis was amply provided for, in the new articulation; although the principal support was derived from the great strength of the capsule.

Case 9.—*Fractured Cervix Femoris—Immediate Shortening—at first slight Inversion, afterwards Eversion of the Limb.*

E. B. æt. 79, fell on the left trochanter. Next morning the limb was three inches shorter than its fellow. At first the toes inclined inwards—subsequently outwards. She was placed on the inclined plane. In two months, the fracture appeared to be united. In two months more, she could bear nearly the weight of the whole body on the limb. In three months more she left the infirmary.

Such is the contribution of Mr. Howship. As we observed in the commencement, it is possessed of much pathological interest. It goes very far to confirm the opinion that, in the great majority of cases of fracture of the neck of the femur within the capsule, no union, or ligamentous union only, is procured. When we consider that the subjects of this accident are usually very old persons, and weigh the small chance of obtaining bony union, use what endeavours we may, we shall be led to agree with Sir Astley Cooper, in not pushing the attempt to an injurious extent. Old persons cannot be long confined, with impunity, to bed. They are apt to fall into a typhoid state, from which they cannot be rescued. Let the surgeon, then, beware how he pursues the phantom of ossific union, to the prejudice of his patient's powers.

We will not indulge in any further observations on the paper we now quit. We recommend its facts to the attention of surgeons, and they cannot but be deemed an useful contribution to the scientific stock that previously existed.

### VI. Observations on Fractures of the Bones of the Pelvis. By Henry Earle, F.R.S. President of the Society.

Fractures and dislocations of the bones of the pelvis are, as Mr. Earle observes, to be looked upon as dangerous accidents, in consequence of the injury likely to be inflicted on the pelvic viscera. Fractures of these bones are also serious, because the violence necessary to occasion the injury must have been considerable. Mr. Earle's object in offering the present paper to the Society is to throw light upon the diagnosis. We will first advert to his cases.

*Case* 1. A man, æt. 40, was admitted into St. Bartholomew's Hospital, in Oct. 1829, with a fracture of the pelvis, occasioned by a fall from a height of 30 feet on the left side. He had lost all control over the left lower extremity, which was everted, but not shortened. Any attempt to rotate the limb caused great pain, and was accompanied with very sensible crepitus when the hand was applied over the hip-joint. On applying the two hands to either side, with a view to compare the relative position of the trochanters with the anterior spines of the ilia, that on the left side was not nearly so prominent as that on the sound side; it appeared, indeed, nearly on a level with the anterior spine of the ilium, and could with difficulty be felt. On pressing the trochanters the patient complained of deep-seated pain in the hip-joint. The patient was placed on a double-inclined bed, a broad leather bandage was passed round the pelvis and buckled firmly in front, and the feet were secured to the foot-board. Subsequently a bandage was applied round the upper part of the thigh, to press upon the trochanter, and to support the whole more firmly. A catheter was passed, but no injury had been sustained by the bladder or urethra. It was necessary, however, to employ the catheter for some time, as the patient could not empty his bladder without it.

The fracture went on well, and in eight weeks after the receipt of the injury, the patient was dismissed, being able to walk nearly as well as before. He died soon afterwards in St. George's Infirmary, of disease of the lungs, and an opportunity was obtained of examining the injured parts.

It was found that the fracture extended in two directions through the acetabulum; that there was an extensive comminuted fracture of the ilium, with some displacement; and that the os pubis was broken in three places. The reparation was complete, and though there was an abundant deposit of callus elsewhere, no considerable quantity was, providentially, thrown out within the acetabulum.

The second case displays no striking peculiarity. The same general symptoms existed in it as in the first, and in both the limb was slightly everted, and the trochanter not so prominent as natural. Mr. Earle's remarks are brief.

"The symptoms in both these cases very closely resembled each other; in both, the nature of the injury was the same, namely, a fall on the side. In both, the most marked symptoms were the *loss of prominence of the trochanter*, and the freedom of motion of the joint, particularly of abduction, which is always attended with the severest pain in fractures of the neck of the femur. The nature of the accident was that which most commonly produces fracture of the neck; the total loss of power over the limb, and the eversion of the foot, resembled what occurs after that accident; but there was no sensible degree of shortening, there was greater freedom of motion, and, instead of the trochanter being more elevated and prominent, as in fracture, it was below its natural level when compared with the healthy limb." 255.

We conceive that no surgeon of moderate experience, or ordinary judgment, could have taken these cases for any thing but what they were, and that there is too little similarity between their symptoms and those of fractured neck of the femur, to permit any, save a raw student, or a member of the noodle family, to commit a blunder. In these cases, there was no shortening of the limb, and the motions of rotation and general opposition must necessarily have been more free than they could be in fractured neck of the thigh-bone. Independently of this, the *great violence* rendered fracture of the os innominata probable, and fracture of that bone was distinguishable by direct examination.

The following case is interesting, physiologically as well as practically.

*Case.* In the Winter of 1833, Mr. Earle was called to a man between 60 and 70 years of age, who had been riding a young colt without a saddle. By a sudden start of the colt, he was forcibly thrown forwards upon the withers. He experienced very severe pain, was unable to retain his seat, and fell from the horse. When Mr. Earle first saw him, he was lying upon a bed in a very faint, exhausted state, and appeared like a patient dying from internal hemorrhage. There was no external wound, but blood was flowing freely from his anus, and had run through the bed on to the floor. There was great flatness of the pubes, and the pelvis appeared broader than natural, allowing a greater separation of the upper part of the thighs. On placing his hand on the front of the pubes, Mr. Earle found no resistance, and over the symphysis and behind the scrotum, extending to the perinæum, there was extensive effusion of blood. Mr. Earle conceived that the ossa pubis were fractured, and apprehending effusion of urine, he carefully introduced a full-sized catheter, which, on arriving at the bulb, took a direction much to the right side, and then entered a great cavity, evidently not the bladder. Mr. E. immediately made a free incision in the perinæum, and let out a large quantity of blood and urine. On introducing his finger, he found that the symphysis pubis had been torn asunder to a great extent, and he felt a smooth projecting body, which at first he supposed to be the distended bladder; but, on more accurate examination, he found it to be the internal surface of the bladder, into which his finger readily entered, and that the prominence was caused by the pressure of the intestines above. On tracing his finger over the surface, the moment he reached the trigone, the patient exclaimed that he wanted to make water. On repeating the contact of his finger with this spot, he was again most urgent to be allowed to make water. The patient lived forty hours, and, on the following day, Mr. E. again gently introduced his finger and touched the trigone, when he again was most desirous of passing water.

On examination after death, the symphysis pubis was separated to the extent of three inches, and the sacro-iliac symphysis on the left side was nearly separated, and gaped to the extent of more than an inch. The prostate gland had been torn away from the bladder, leaving a large aperture communicating directly with the cavity of that viscus. The urethra still retained its connexion with the ligament on the right side of the pubes, and the prostate gland hung loose in a cavity filled with coagulum. An extensive laceration communicated with the rectum.

Mr. Earle very properly draws attention to the necessity of examining the condition of the bladder by the catheter in every case of fracture of the pelvis. Laceration of the bladder or urethra is too common a complication of this kind of fracture. We have ourselves seen several instances of this fact, and a very serious state of things it is. Mr. Earle remarks, and the caution should be deeply impressed upon the minds of young or inexperienced surgeons:—

"I take this opportunity of strongly urging the propriety of a cautious examination of the urethra in every case of suspected fracture of the pelvis: next to extensive internal bleeding, the most alarming and certainly fatal occurrence, is effusion of urine. The former it may not be in our power to control or obviate, but the latter we may often prevent, and by timely assistance, save the patient. Whenever it is clearly ascertained that the urethra is ruptured, and the catheter cannot be passed into the bladder, it will be right at once to make a free incision in the perineum, and thus allow of a free exit for the urine. It fortunately happens in many cases that the effused blood compresses the urethra and prevents the escape of the urine, and thus time is allowed for taking the necessary steps, but these should on no account be delayed.

In many doubtful cases of fracture of the pelvis, an examination with the finger per anum will enable the surgeon to detect the nature and extent of the injury." 264.

A physiological point remains. In the case which we have just related, the application of the finger to the trigone produced each time an urgent desire to make water. This is an interesting fact, and bears out completely the opinion previously entertained by many, that this was the sensitive part of the bladder, the part that operates in producing many of the physiological phenomena displayed in the excretion of the urine, and the sympathies of the bladder and the kidneys.

" It must be admitted," says our author, and with this passage we leave him, " that this opinion receives much confirmation from the well known facts, that when a catheter or other instrument is introduced into the bladder, so as to touch this part, a distressing urgency to pass water is experienced, even when that viscus is empty, and still further by the circumstance, that when a stone is in contact with this part the same urgency is experienced, which is relieved by change of posture and consequent removal of the stone from pressing on this sensitive spot. It is true that these facts have been adduced by authors favouring such an opinion as has been stated above, but in the present case I had the strongest confirmation of the peculiar sensibility of this part, as I repeatedly traced my finger over the other parts of the bladder without causing any particular uneasiness, but the moment I touched the trigone the patient most urgently entreated to be allowed to make water; thus affording the strongest possible evidence of the correctness of the opinion that this portion is endued with a peculiar degree of sensibility. I was enabled also to ascertain that the mucous membrane at this part remains quite smooth when the rest of the bladder is thrown into folds." 261.

### VII. Cases and Observations illustrative of Diagnosis when Adhesions have taken place in the Peritoneum, with Remarks upon some other Changes of that Membrane. By Richard Bright, M.D. F.R.S. &c.

Dr. Bright commences with an observation highly philosophical in itself, and with which we cordially agree—that any means of improving diagnosis, whether attended with immediate apparent utility or not, must be satisfactory to the physician. We all know that a certain class of persons exist, not only in our own, but in all professions, and not only in professions, but in every class of society, who constitute the drags on the social and the intellectual wheel. These gentlemen are ever exclaiming " stop." In medicine they are especially averse to pathology—it is too methodical, too exact—too little consistent with the good old notions and the good old practice—the good old practice and the good old notions of the dogmatists and the empirics. If you shew these gentlemen that inflammation of the lungs consists in certain changes of a certain texture—if you prove to them that what they always believed to be a very common occurrence, the formation of an abscess in the pulmonary parenchyma, is really extremely rare, they shrug their shoulders, and exclaim " cui bono ?" It is true that exact knowledge of the alterations in which disease consists, or which are its immediate consequences, does not tell you on the instant a new remedy, or enable you at once to cure what was formerly incurable; but it does tell you what you are about—it does tell you when to act and when to stop—it gives precision to your practice and opinions—and it enables you to apply, with your eyes open, the remedies you formerly wielded in the dark. Would one of those gentlemen like to have his information less exact on any of his personal affairs—would he think that his management of his funded or his landed property could be made one whit the better, by his being reduced to a happy state of doubt on its precise condition? We will venture to say that he would not—we will venture to say that no man out of Bedlam could contend, as a general position, that the affairs of life are better conducted, the less definite our knowledge of what appertains to them is. In our science, much must ever be a matter of doubtful calculation. Surely what tends to diminish that doubt, must sooner or later, directly or remotely, improve the art in which uncertainty constitutes the material difficulty.

The gist of Dr. Bright's communication is contained in the following explanatory passage.

"I shall then content myself with stating, as a sufficient preface to most of the cases contained in the following communication, that I have observed on several occasions, that when the circumstances of the disease had rendered it probable *that adhesions might take place between the viscera and the peritoneum of the abdomen, a very peculiar sensation has been communicated to the touch, varying between the crepitation, produced by emphysema and the sensation derived from bending new leather in the hand.* And in each of the cases which I shall now detail, I have had the opportunity of discovering, by examination after death, that such adhesions had existed in the parts where this sensation was discoverable; whereas, in no case have I observed the phenomenon, and ascertained that the particular morbid condition did not exist; so that I am led to infer the probability that the same adhesive process had taken place in those cases where no opportunity of post-mortem examination was afforded." 178.

Dr. Bright relates seven cases, with the addition of the memorandum of another supplied to him by Mr. Copland Hutchison. The cases are detailed with some prolixity, and it is quite unnecessary for us to go into their several particulars. We shall therefore select those which prove, or seem to prove, the poinst insisted on.

*Case.* A sea-faring man, æt. 42, was admitted into Guy's Hospital under the care of Dr. Bright, on the 25th of March, 1829, with ascites and anasarca. He stated that thirteen months previously he had been attacked with inflammation of the liver, succeeded by accumulation of fluid in the abdomen—that this subsided, but three months before his admission returned. Six weeks before his admission he was tapped, and seven quarts of bloody serum were drawn off, with little diminution in his apparent size.

On the 1st of April, he was again tapped by order of Dr. Bright. Four gallons and two quarts of serum, highly tinged with bile and coloured with blood, were drawn off. The abdomen rapidly filled again. On the night of the 19th, he required to be cupped for severe pain in the left side. In the beginning of May he had dysenteric symptoms. On the 19th of June he was again seized with severe pain on the right side of the abdomen; it was relieved by bleeding and calomel and opium. After this the abdominal effusion decreased.

July 8th. The abdomen, though greatly diminished and flaccid, is still projecting, and communicates to the feel the sensation of a semi-solid substance, without fluid, *and gives to the hand an impression as if two surfaces passed over each other, with adhesions between them;* it is a *kind of crepitus.* Though quite free from pain he evidently loses strength, and his face grows thin and sallow.

Diarrhœa came on, and on the 30th the patient sank.

*Sectio Cadaveris.* " The body was much emaciated, the abdomen large, particularly in the central part, below the umbilicus. On laying open the parietes of the abdomen, not above one or two pints of serous fluid escaped. *On the lower part, and particularly below the umbilicus, and to the right side, strong adhesions had formed between the peritoneum of the parietes and a large tumour:* and on separating the attachments, which could scarcely be effected without laceration, it appeared that the tumour was a mass of fungoid substance, bearing in some parts a lobulated character, but every where soft and spongy to the feel, attached to the stomach, and descending quite to the pelvis. It was slightly glued to the viscera by adhesions, which were evidently of late formation, and were easily torn through. When the whole mass was raised, the intestines, both small and large, were found lying behind it, chiefly pressed into the upper part of the lumbar region, and under the margin of the ribs. On examining more carefully its connexions above, it was found firmly united to the right half of the large curvature of the stomach, and to the omentum, which lay as a fatty mass, not very unhealthy in appearance, over the upper part, and besides the tumour, was the only object which came into view when the abdomen was first opened. The stomach felt remarkably hard and solid over the whole of its pyloric half; and on opening it, a mass of lobulated projections was seen rising from the large curvature, filling up one-half of its cavity. The mucous membrane covering this was in part healthy; but at the apex of each lobule might be seen a depression, as if ulceration were beginning to take place; and in one part, about the centre of the mass, an oval portion, larger than a shilling, was entirely abraded. The pylorus itself was pretty free from disease.

The structure of the tumour was very peculiar. It was surrounded by an investing membrane, on which many large vessels were distributed; and in some parts it felt softer and more fluid than in others, rising in these parts into semiglobular elevations. On cutting into it, the greater part was found to be composed of a fibrinous mass, not unlike the coagulum of blood; in some parts more red, in others less so, and in a few parts membranous cysts of considerable strength were found, containing bloody fluid. The whole tore easily as soon as the external membrane was divided." 182.

The intestines were drawn together by adventitious membranes. The other morbid appearances are not deserving of attention.

Before we proceed, we may observe that a stranger, a non-professional stranger we mean, reading a case of this description, would think it rather odd that a doctor should dwell on the discovery of a few unimportant adhesions between the integuments and a tumor, when the tumor itself would seem to have escaped observation. In his ignorance, he would suppose that this was, indeed, to strain at a gnat and swallow a camel. And we must say it does seem singular that so large a tumor, involving the stomach so materially and so extensively, should not have been detected during life. But this is par parenthèse. We will give another case—our author's fourth.

*Case.* " In April, 1832, I was requested to meet Dr. Pidduck and Dr. James Clark, in consultation in the case of a gentleman, who after severe illness had become the subject of ascites, which had now existed in a very decided form about two months. I need not at present enter minutely into the circumstances of the case; it is sufficient to say that it was thought right to perform the operation of paracentesis, and above four gallons of clear, pale, straw-coloured serum were drawn off by Mr. George Babington. The operation was repeated in a fortnight, when three gallons of a fluid rather more yellow than the last came away. Two days after the second operation, I noted that a *slight sensation of crepitation was distinctly felt over the situation corresponding to the liver and omentum.* This was perceived but for two or three days, and then became indistinct.

Ten days after, seven quarts of clear fluid were drawn off, and the abdomen, though all the fluid seemed to be taken away, was not diminished to its former size, and a distinct hardness was now felt about the situation of the left lobe of the liver, which was attributed to a corrugated state of the omentum. Nearly six weeks now elapsed before the operation was repeated, when one gallon of fluid, of a light colour, was evacuated." 192.

After this operation the fluid did not re-accumulate, but in June, 1834, the patient died.

*Sectio Cadaveris.* The whole abdomen covered with fat, at least two inches in some parts. About two pints of fluid in the cavity. The *colon and omentum were adherent, in one mass, to the parietes of the abdomen.* The liver was lying under the ribs, concealed at first by the mass of corrugated fat, omentum, and adhesive matter. When the liver was a little drawn down, a hard and almost cartilaginous coating, with which the right lobe was covered, separated from it. The small intestines were quite free from adhesion. The liver was small, and a well-marked specimen of what is termed the " hob-nail liver."

Dr. Bright suspects that, if the adhesive inflammation could be excited in the peritoneum, even short of the extent of producing actual adhesions, the effusion of serum would be prevented. The suspicion is as ancient as the time of Sir James Earle at the very least, for it was on this very supposition that injections were used for the cure of hydrocele, a dropsy of another serous membrane.* Attempts have been made in many ways to apply this principle, or rather fact, to the cure of dropsy. A canula has been left projecting into the abdominal cavity, after the operation of paracentesis—tight bandages have been employed—nay, even injections have been proposed. These means have failed, and probably will ever fail, for two good reasons —first, the cavity in which they are used is so extensive, that they are productive of serious danger—and, secondly, the dropsy is in general the effect of organic visceral changes, beyond the reach of applications to the peritoneal surface. As Dr. Bright's speculation, then, is not new, neither, we fear, are his anticipations likely to be gratified.

Dr. Bright remarks that the crepitation, which would seem to be a symptom of abdominal adhesions, does not continue for any length of time. It appears to belong, though by no means exclusively, to some early period of the formation of adhesion, probably before the contraction of the newly-formed fibres has occurred. Our readers are now in possession of this contribution towards diagnosis, and it remains for the profession to test its general accuracy and value.

Dr. Bright concludes his paper by a sketch of the most remarkable mor-

---

* It is well known to surgeons, that hydrocele is occasionally cured by the injection, although no actual adhesions have occurred.

bid conditions of the peritoneum, which he has observed more particularly in connexion with ascites. The study of these is calculated to exhibit the general and local signs of adhesions, independent of that particular crepitation on which we have been dwelling.

"One of the most frequent morbid changes in the peritoneum, is when the whole is covered with an equally distributed false membrane, which renders it, in its general appearance, opake, and is apt to contract the loose folds of the membrane and those by which the various viscera are suspended or attached, and likewise to form a kind of compressing ligature about all the viscera themselves; the result of which is, that the omentum gradually becomes shortened and corrugated, ultimately forming but a narrow band along the arch of the stomach and the colon,—the mesentery becomes shortened, and the intestines, by this means, drawn towards the spine,—the calibre of the intestines themselves becomes diminished, and they are most obviously shortened in their course,—the liver is drawn close to the diaphragm and the spleen to the stomach,—while the kidneys are fixed more firmly into the cavities formed by the muscles of the loins,—and all these viscera are compressed in a degree which often produces alteration in their shape, and decidedly interferes with the integrity of their respective functions." 196.

This false membrane is polished, and looks like peritoneum; but it is really only lymph, and admits of being stripped off in flakes.

Dr. Bright looks on this as the product of a very low stage of chronic inflammation. The only signs, he says, by which it is to be ascertained are, the general derangement of the various functions, and the circumstance, that on employing percussion while the abdomen is still full of fluid, the superior part, as the patient lies on the back, yields no hollow sound,—which is generally the case when the intestines are free to float upon the surface of the serum, but which, now that they are held back towards the spine, cannot be perceived. We should be sorry to trust to a sign so vague, that in the eye of practical philosophy it cannot be considered a sign at all. A contribution to a guess it may be—a physical sign it is not. The best sign is in the general symptoms—the slight fever—the dull pains, increased on pressure, and aggravated by active exertions—the impeded or the altered functions; and yet it must be owned that, even with great care, diagnosis is difficult, and, without great care, it is exceedingly precarious.

"This adventitious covering of the peritoneum (continues our author) has occasionally been found thickened to so great a degree as to obscure the sense of fluctuation, and convey the idea that the fluid was encysted, when in fact it has been in the peritoneum itself. I once had a case under my care, in which a female was tapped fourteen times, without suffering any untoward symptoms, the fluid re-accumulating in general very slowly, and, on one occasion, remaining so long absent, as to inspire the hope that a complete stop had been put to the effusion. When the fluid was drawn off, the general impression remained of a large empty cyst, and a hard ovarian tumour was distinctly felt rising from the left side of the pelvis; but when, after a lapse of nearly three years, she died, the diseased ovary was indeed found, but it was clearly ascertained that the fluid had been drawn from the peritoneal cavity, and so thick was the false membrane deposited on that portion which lined the parietes, that it resembled, in some parts, a layer of cartilaginous matter." 198.

This case he afterwards details at length; but the abridged remarks contain the sum of its useful features. To proceed with the alterations of the peritoneum:—The deposit of adventitious matter, though frequently universally and equally distributed, is sometimes found to assume more diversity of appearance, and more particularly on the convex surfaces of the spleen and liver it is remarkable for its honey-combed arrangement; a circumstance not improbably depending upon the interruption which is received during its deposit, by the sliding of their surfaces over the neighbouring surfaces of the diaphragm or ribs, and the interrupted effort at forming adhesions to their adjacent parts.

"Many of the appearances which present themselves on the peritoneum seem to depend upon alterations taking place in this adventitious membrane, the feeble organization of which probably renders it liable to morbid changes and the development of morbid growths. Amongst these appearances is that of a carbonaceous deposit, more or less extensive, and varying in the intensity of its colour, and which I conceive to be the result of extravasation of blood into the newly-formed tissue, under some peculiar circumstances as yet not sufficiently understood;—I say, under circumstances not sufficiently understood, because we do not know even whether it be the peculiar nature of the blood, or the peculiar structure of the part into which it is effused, which (supposing it to arise from effused blood at all) gives the liability to this black or gray appearance, to which I am perhaps premature even in ascribing a carbonaceous character. This black deposit has in its appearance a striking resemblance to the black, pulmonary matter formed in the tissue of the lungs; and it seems to be of the same nature as that which forms the minute speckling of gray on the internal surface of the intestines, when they have been subject to continued chronic irritations. I have likewise seen it on the membranes of the brain, in cases where death having occurred from recent effusion of blood, rendered it probable that the black appearance was the result of similar extravasation at a former period.

This gray matter is variously distributed on the peritoneum. I have seen it in patches about the lower part of that which lines the parietes, and in other parts of that portion of the membrane; I have also seen it in the pelvis, and frequently on the surface of the intestines themselves; and I shall now relate a case which has lately occurred to me, which seems to shew its connexion with an hæmorrhagic tendency most probably taking place in the newly-formed vessels of the adventitious membrane." 203.

Dr. Bright relates a case in illustration of the preceding observations. It is indeed very difficult to determine the precise cause of these black deposits. We have seen them exist concurrently in the peritoneum and the pulmonary and costal pleura; and we have seen them where there was no adventitious tissue, or mark of inflammation of the serous membranes. In a case which lately occurred at St. George's Hospital, and of the history of which we are ignorant, the peritoneum and the pleura were universally studded with small black patches, giving the membrane a speckled appearance, not unlike that of the integument of some reptiles. But in neither the pleura nor the peritoneum, were there, so far as we can remember, any signs of inflammatory action. Whether this carbonaceous deposit depends or does not on alterations of blood effused, must yet, we conceive, be looked on as a very doubtful point.

Passing over the case detailed by Dr. Bright, we proceed with his pathological remarks.

He points out the tendency to internal hæmorrhage, occasionally exhibited in cases of ascites. It often happens that where the fluid drawn in ascites has, during several operations, been quite free from blood, the succeeding operations are attended by the discharge of highly tinged serum. This condition, and that in which the serum has a deep tinge of bile are by far the most frequent, if not the only peculiarities of appearance, which the serum of the peritoneum acquires in genuine ascites, even where it is connected with much disease of a malignant character; so that, when in doubtful cases of ovarian dropsy, (cases rendered doubtful by the extent of the cyst, by the general fluctuation, and by the absence, at least during the distended state, of any perceptible secondary tumours,) we find the fluid assuming either the opake appearance of puriform matter, or a ropy consistence, or the grumous appearance of long deposited and altered blood, we may, Dr. Bright thinks, generally conclude in the absence of other proofs, that the fluid is not secreted by the peritoneum. Our author observes also, that he has never seen in the fluid of genuine ascites, that deposit of cholesterine, which Dr. Bostock has described. This appears to take place generally when the fluid has been long collected in cavities, under different circumstances of disease, and amongst these the ovarian cyst may not unfrequently be ranked. It is not unfrequently remarked in the fluid of hydrocele. We have seen the crystals more than once in the liquid removed by the trocar.

We return again to the subject of adhesions, and their diagnosis. When they exist only to a small extent, their diagnosis is mere conjecture. They may or they may not interfere with some important function. An irregular band may occasion habitual constipation or actual strangulation. Few experienced physicians or surgeons have failed at one time or other of their lives to witness an instance of this description. But we repeat that the diagnosis is little more than chance. A greater amount of adhesions afford more likelihood of giving birth to tangible signs. Dr. Bright is rather sanguine.

"But when," he says, "the adhesion has been more general between the viscera, or the convolutions of the intestines, a peculiar feeling of hardness is communicated to the touch, over a greater or less extent, which awakens in the experienced physician strong suspicion of the fact; and when this has become universal, matting the intestines into one mass, the hard, unyielding feel which is discovered, or the dough-like sensation which is felt, or the unnatural, rounded mass which fills the place of the small intestines, present almost convincing proof of the condition of the peritoneum. Should there be, in addition to this, a want of that sensation of the viscera slipping from beneath the touch which is recognized in the healthy state of these parts, the probability is, that whether the viscera adhere amongst themselves or not, they are likewise glued to the parietes; and if still further, or if without the other symptoms, that peculiar *crepitus* of which I have spoken should exist, scarcely a doubt would be left in my mind that adhesions had formed." 208.

The mode of adhesion varies very greatly with the severity of the inflammation and the constitutional peculiarity of the patient. It may be simple lymph—or formed of fine bands and meshes, resembling cellular membrane—or every variety of morbid deposit may occur in it, scrofulous matter, or that of scirrhus, or medullary sarcoma. Dr. Bright supposes, with probability, that the first kind of adhesions alone (that uncomplicated with morbid deposits) gives rise to the diagnostic crepitus.

When morbid alterations of structure are deposited in the adventitious membranes, they are apt, as Dr. Bright observes, to induce a still greater degree of hardness and of solidity in the diseased portions of the abdomen, and when they go to a great extent, present a board-like resistance to the hand, which no relaxation of the abdominal muscles can remove; and which becomes still more remarkable if the viscera are glued to the parietes, in which case we may often, as the patient becomes emaciated, discover by the feel something of the nature of the cementing deposit.

There are no doubt cases in which an accurate opinion can be formed from data of this character. But the practical man is compelled to own that the discrimination of abdominal tumors is in many instances extremely difficult, and that in a consultation the conclusions formed from manual examination are frequently as numerous as the consultants. Such is unfortunately too often the case, but we say it tends to stimulate rather than discourage. With the following additional suggestions of our author we terminate our notice of his communication. He relates one or two other cases, but we do not think they carry any additional evidence of consequence.

"It is indeed," he owns, "often totally impossible to determine, from local symptoms, whether this deposit be scrofulous and tubercular or malignant in character. Yet it sometimes happens, more particularly when, by tapping, any inflammatory action has been induced in the cellular membrane of the integuments, that they assume a character at the part where the adhesion takes place, which is by no means equivocal; and in illustration of this point, I may hereafter offer some cases, shewing that occasionally, where the internal disease has been of a malignant character, the neighbouring parietes have indicated the fact by pretty speedily becoming the seat of true scirrhous tubercles, felt like small beans or peas under the hand, and quite superficially, as I have likewise more than once had occasion to observe, on the parietes of the thorax, where malignant adhesion has taken place between the lung and the pleura costalis. When the deposit is of a tubercular character, we frequently have a granulated feel communicated to the spread hand applied over the abdomen, and we generally discover other symptoms of the tubercular diathesis by which our suspicions are awakened." 210.

When, as sometimes happens, the patient has previously exhibited elsewhere appreciable malignant disease, the diagnosis of the visceral tumor is of course facilitated. Something also may be gained by a close examination of the constitutional condition and peculiarities. This paper, like all of Dr. Bright's, is ingenious, valuable, and worthy of attention.

### On Affections of the Brain.

I. On Serous Effusion from the Membranes, and into the Ventricles of the Brain, and its connexion with Apoplexy, and other Diseases of the Brain. By John Sims, M.D. &c. &c.

II. On Hypertrophy and Atrophy of the Brain. By J. Sims, M.D. &c.

III. On the Cure of Ramollissement of the Brain. By J. Sims, M.D. &c.

IV. A Relation of some Cases of Mental Derangement successfully treated by the Acetate of Morphia. By E. J. Seymour, M.D. &c.

We throw together these four papers, but not for the purpose of amalgamating them. They treat of different affections of the cerebrum, and are so far different things, but still they do treat of the cerebrum, and we therefore place them in a connected series. We shall take them in the order in which we have arranged their titles.

The object of Dr. Sims' first paper, that on serous effusion within the cranium, appears to be to prove that what is commonly called serous apoplexy, either has no existence or is extremely rare, and that the fluid discovered in or on the brains of those who die suddenly, is not to be regarded as the essential cause of their sudden death. This seems to be the gist of the following facts and observations.

Dr. S. sets out with the broad statement, that on dissecting the bodies of persons who have died of apoplexy, three morbid states of the brain have been more particularly noticed:—an unusually loaded state of the blood-vessels,—extravasation of blood,—effusion of serous fluid from the membranes or the ventricles. Other morbid changes have been found, as tumors, diseased blood-vessels, cysts, and so forth, but they are, to use the language of logic, the accidental modes, and the three pathological states first mentioned are regarded as the essential ones. He proceeds to observe that, while systematic authors have divided apoplexy into two species—the sanguineous and serous—and have attempted to define the symptoms and the characters of each; many practical writers have doubted the frequency of the serous variety, or denied its existence altogether. Disregarding the opinions of the older anatomists, he quotes the more modern British authors—Heberden—Cheyne—Haslam—Crowther—Mills—Cooke—Bright—and Abercrombie.

The quotations of our author exhibit a tolerably decided leaning on the part of some or of all these gentlemen against the opinion that makes serous apoplexy a common form of disease. But Dr. Sims observes in conclusion that, notwithstanding the preceding evidence, it is still a very prevalent opinion, that if a person, rather advanced in life, fall down in a fit, and suddenly expire, or survive but a short time with coma, insensibility, stertorous breathing, &c.—and that if the head of such a person be examined, and effusion to any extent be found between the membranes or in the ventricles without any extravasation of blood, the death has been occasioned by what is termed serous apoplexy. This opinion which he considers prevalent, it is the object of his paper to prove erroneous. His cases and his observations, he thinks, will lead to the conclusion, that persons dying suddenly or speedily under the circumstances mentioned above, are more likely to have suffered an attack of simple sanguineous apoplexy, or that form of the disease in which a loaded state of the blood-vessels is discovered on dissection. His cases and remarks are detailed and classified under five heads:

I. Serous effusion of the brain, or its membranes, of persons dying of various diseases not cerebral, and who had manifested no symptoms referable to the brain.

II. Serous effusion into the ventricles or membranes, to a considerable extent, in cases where old apoplectic cysts were found, with or without attendant paralysis: the patients being destroyed by disease not cerebral.

III. Serous effusion into the ventricles or membranes of unquestionably long standing, with old apoplectic cysts: the patients being destroyed by recent extravasation of blood within the cranium.

IV. Cases of simple sanguineous apoplexy.

V. Cases of serous effusion into the membranes or ventricles of old standing, with loaded, dilated, or diseased bloodvessels, frequently denominated serous apoplexy, but more properly referable to simple sanguineous apoplexy.

We now start fair. Dr. Sims' intentions are laid open to our readers, and we have only to see how he executes his task.

1. *Serous Effusion in the Brain or its Membranes in Persons dying of various Diseases not Cerebral, and who manifested no Symptoms referable to the Brain.*

It is an observation which must occur to, and is daily made by every practical physician or surgeon, that effusion, especially into the sub-arachnoid cellular tissue, is very frequently observed in the examination of dead bodies, where no cerebral symptoms existed during life. The observation we say is so common, as almost to amount to a truism. Dr. Sims gives a table containing fifty cases of persons who died of various diseases not cerebral, and who manifested no symptoms referable to the brain, but exhibited in it or in its membranes on dissection, effusion of fluid and other morbid appearances. The table itself is too long for insertion, but the following are abstracts of its most important facts.

1. Effusion of serous fluid into the cavity of the arachnoid tissue, or both, was observed in considerable quantity in nearly all the cases: in many of them several ounces were found in the ventricles.

2. A loaded or diseased state of the bloodvessels—opacity and thickening of the membranes—with other morbid states, were noticed in many of the cases.

3. The respective ages of the subjects were—

| | | | |
|---|---|---|---|
| Under | 10 years | | 4 cases. |
| Between | 10 years and 20 years | | 2 |
| | 20 | 30 | 8 |
| | 30 | 40 | 4 |
| | 40 | 50 | 5 |
| | 50 | 60 | 12 |
| | 60 | 70 | 10 |
| Upwards of 70 | | | 5 |

It appears that between the ages of 50 and 70 nearly one half of the cases occurred.

4. Of the diseases which occasioned death, there were of—

| | |
|---|---|
| Phthisis pulmonalis | 16 cases. |
| Disease of the heart | 8 |
| Purpura hæmorrhagica | 1 |
| Cholera | 1 |
| Carcinoma and fungoid disease of stomach, liver, kidney, &c. | 9 |
| Pneumonia | 3 |
| Peritonitis | 2 |
| Phlebitis | 1 |
| Crural phlebitis | 1 |
| Inflammation of liver and peritoneum | 1 |
| Mortification | 1 |
| Diseased bladder | 1 |

| | |
|---|---|
| Ulceration of intestines .................. | 3 cases |
| Internal abscess ......................... | 1 |
| Scrofulous joints ......................... | 1. |

As Dr. Sims observes, it is not surprising that the majority of cases should be instances of phthisis, that being the disease of which most die. But Dr. Sims makes another remark, the justice of which we question, and the premises of which we do not see. Any one, he says, accustomed to pathological investigations, must have observed how very frequently (more especially in the latter periods of life) disease exists in the muscular structure of the heart, and in the mitral and tricuspid valves, without having produced any notable inconvenience, and very frequently without having been discovered during life: it would therefore appear that human life is not shortened, in many instances, by very extensively diseased states of the heart.

Dr. Sims is attached to an institution, the majority of whose inmates, as the majority of his patients, are old people. In a workhouse, it must be difficult, indeed impossible, to determine the causes of death in the middle periods of life. The very young and the very old are the tenants of the poor-house. The active and middle-aged form the patients of hospitals. In them, we can best determine whether diseases of the heart materially shorten life. We have no hesitation in expressing our opinion that they do. The majority of those who die in hospitals, between the ages of 40 and of 50, die of diseased heart. Dropsy is more frequently a consequence of it than of hepatic alterations; and how fatal dropsy is, we need not tell.

It may be said that disease of the heart is found in the bodies of very old persons, and that, therefore, it cannot have materially shortened life in them. That is true, but we must not take the observation for more than it is worth. Disease of the heart is, in fact, the disease of the later periods of life—it is the usual means that Nature takes to close existence. There are comparatively few aged persons, in whom the left ventricle is not found hypertrophied, and the arteries more or less altered. Abstract these changes, and observe whether the other natural alterations would materially tend, in the majority of instances, to put out the individual. They would not. It is the changes in the circulating system, that more than any others limit the duration of existence. We cannot, therefore, agree with Dr. Sims, that life is not shortened, in many instances, by very extensively diseased states of the heart. To prove that position, it is necessary to establish that the person would have died as soon, had no disease of the heart occurred. A little reflection would shew that this is difficult. The practical physician looks on alterations of the heart with dread. It is for them that, after the middle periods of life, he looks, and looks with suspicion and with dread. What a false security there would be for him and for his patient, if he thought that extensive disease of the heart was often comparatively immaterial.

A review of the summary already given of the principal facts contained in the table, shews that the greater number of the patients in whom serous effusion was found, unaccompanied with cerebral symptoms during life, were old persons—and shews, too, that the effusion was principally seated in the cavity of the arachnoid, or the cellular tissue beneath it. Our own dissections would lead us to believe, that arachnoid effusion may be regarded as almost a natural change—in other words, that it is generally found to exist in persons advanced in life, under all varieties of circumstances. In the number of this Journal for September, 1829, is an account of some dissections, in which arachnoid effusion was found. The cases were reported by the junior Editor of this Journal, for the purpose of shewing that such effusion was probably independent of cerebral symptoms, or, in round terms, of cerebral disease. Since that time, further investigations have made suspicion amount to certainty, and have convinced us that such effusion in such cases, and, indeed, in many others, must be looked on as a common appearance, not necessarily connected with material cerebral disorder.

2. *Serous Effusion into the Ventricles or Membranes to a considerable Extent, in Cases where old Apoplectic Cysts were found, with or without attendant Paralysis, the Patients being destroyed by Diseases not Cerebral.*

Four cases are related. They present little or nothing to detain us. They prove, what no well-informed man denies, that in cases of old apoplectic extravasations, or of other organic changes in the brain, patients may die from other causes, and after death serous effusion shall be found, which must have long existed, yet seems to have occasioned no special symptoms.

3. *Serous Effusion into the Ventricles or Membranes, of unquestionably long Standing, and old Apoplectic Cysts, with recent Extravasation of Blood, which speedily destroyed Life.*

The following short case will serve for an illustration of this section. Five others are related, but require no especial notice.

*Case.* " Thomas Dalton, æt. 50, six feet high, has amaurosis of both eyes, which is reported to be consequent on purulent ophthalmia. He was admitted into the insane ward about three weeks ago. During the first part of the time he was quiet, but latterly he was occasionally boisterous, and required restraint. Early in the morning of the 7th instant, one of the medical assistants was called up to him, who found that he had suffered an apoplectic seizure. Signs of approaching dissolution soon came on, he however rallied a little, and lived about thirty hours after the attack.

*Inspection,* 25 *Hours after Death.* Nov. 9th, 1833. Brain.—A large quantity of fluid was discovered between the membranes, and also in the ventricles. Nearly the whole of the arachnoid lining the dura mater on the left side was covered with a layer of coagulated blood, and in a slight degree also on the right side : the layer was thicker at the posterior part of both hemispheres. One of the large veins of the pia mater was distinctly observed to have been ruptured. The vessels of the pia mater and the substance of the brain were unusually loaded with blood. The substance of the brain, particularly the grey matter, was remarkably hard, resembling soft cartilage to the knife. The optic nerves, throughout their course, appeared like flattened cords of filamentous tissue, without any of the ordinary substances of a nerve. The corpora quadrigemina were remarkably diminished in size.

Thorax.—Heart; globular hypertrophy of the left ventricle, but to a very small extent." 293.

Such cases are not rare; and all who have seen much, must have seen many of the same description.

4. *Simple Sanguineous Apoplexy.*

Dr. Sims, before entering on the details of any cases, adverts to the question, whether hyperæmia can occur within the cranium. To this a sufficient answer can be given. Whatever be the compressibility or incompressibility of the brain, we find in pathological investigations very various, we might say opposite, states of its sanguineous system. One brain is pale—another has its venous system loaded—another displays arterial injection. Are not these different conditions, and must not their effects on the cerebral tissue and cerebral functions differ too? Reason would seem to say yes, and experience bears reasoning out.

If we do not admit the efficiency of sanguineous effusion to explain certain marked phenomena, how can we account for such cases as the following?

*Case.* "A. B. æt. 23 years, a stout, muscular young man, six feet, two inches high, very short neck, accustomed to high living, whilst reading was suddenly seized with apoplexy. He screamed out immediately on the attack, and sunk into a state of insensibility, from which he was never roused. He died in the course of a few hours.

*Inspection.*—Head. Scalp highly congested. Skull purple, mottled with blood. Brain: the dura mater adhered to the bone, and the superior part came off with the calvaria. There was slight serous effusion in the subarachnoid tissue, over a few of the intergyral spaces. The blood-vessels throughout the membranes and the substance of the brain were gorged with blood; a large quantity of blood flowed from the incisions made into the sinuses. No extravasation could be detected. There was a small quantity of fluid in the ventricles, and the central parts of the brain were rather soft.

Thorax.—Heart rather large, but natural in structure. The lungs were loaded with blood posteriorly. A large quantity of fluid blood was found in the trachea and ramifications of the bronchi. No laceration of the texture of the lungs or of any blood-vessel could be detected. The capacity of the thorax appeared to be encroached upon by the liver.

Abdomen.—Stomach contracted and rugous, the mucous membrane vascular. The liver was more than twice the natural size, in part from congestion, without any apparent change of texture." 300.

We have now brought before our readers the groundwork of our author's argument—the principles and facts, armed with which he is to encounter and slay the giant prejudice of serous apoplexy. Observe what has been done. Facts have been produced to shew that serous fluid exists, in very many instances, in the brain of persons who died of diseases not cerebral, and who manifested no symptoms of effusion; that fluid is often effused in great quantity in the membranes and ventricles of those persons who have previously suffered apoplexy with extravasation of blood; and that some of these persons live to the ordinary period of human life, being carried off by various disorders, and that others are destroyed by subsequent extravasation of blood in the brain. And, finally, instances of simple sanguineous apoplexy have been related. In short, it has been fully proved, upon the one hand, that serous effusion exists without symptoms, and, on the other, that symptoms are presented, when only sanguineous congestion in the brain can be discovered. Armed thus with facts and with deductions, we pass to—

5. *Cases of Serous Effusion into the Ventricles or Membranes, of old Standing, with loaded, dilated, or diseased Bloodvessels, frequently termed Serous Apoplexy, but more probably referable to simple Sanguineous Apoplexy.*

Five cases are detailed in illustration of this section. We shall take one as a sample of the whole.

Case.—*Copious Effusion into the Ventricles—Great Congestion of Bloodvessels—old Cysts—Atrophy of the Brain.*

William Parker, æt. 62. He suffered an apoplectic fit late in the evening of Aug. 4th, 1834. His face was drawn to the left side; there was paralysis of the right arm and leg. His intellect had become impaired, and he had been confined to bed three or four months past. He has dragged his right leg for twenty years. He lived about 24 hours after the attack.

*Inspection, Aug. 6th, ten Hours after Death.*—Head: Brain. Dura mater was tough, and there were opaque spots on the arachnoid. The bloodvessels were very considerably loaded. There were two cysts of old standing on the superior surface of the right hemisphere, as large as a walnut; the lining was fawn-coloured, and they contained fluid: these cysts did not touch upon the central parts of the hemisphere. A similar cyst existed in the centre of the left hemisphere; the corpora striata and thalami were entire, and appeared healthy. On the surface of the right lobe of the cerebellum there were small remains of an old extravasation. The ventricles were very large, and contained several ounces of fluid. The brain appeared much diminished: weight 2 lbs. 6 oz.

Such are the principal facts upon which Dr. Sims grounds his opinions. Of those opinions, the following is offered as a final summary. Prior to presenting it, he guards himself against the imputation of denying the existence of serous apoplexy altogether; and he does not extend his views to that form of inflammation of the brain in children or adults, termed hydrocephalus acutus—in which a collection of fluid in the ventricles constitutes one of the pathological conditions observed upon dissection. This being premised, we proceed to state the inferences of Dr. Sims:—

"1st. That very copious effusion of serous fluid is found between the membranes and in the brain of persons of all ages, more especially between the ages of fifty and seventy years, who have died of various diseases of the thorax and abdomen, and who manifested no appreciable symptom of cerebral disease during life. That under similar circumstances, various other morbid appearances are found in the brain, as thickened membranes, diseased bloodvessels, tumours, &c. That these several morbid states of the brain did not occasion the death of such persons.

2dly. That effusion of serous fluid into the ventricles and membranes, to a very considerable extent, exists in the brain of persons who have suffered previous apoplectic attacks, attended with paralysis and general or partial atrophy of the brain. That, most probably, this effusion and atrophy have existed for many years, without producing the disease termed serous apoplexy: that persons so circumstanced live to an advanced age, and are destroyed by other maladies.

3dly. That in persons whose brain has long contained a large quantity of fluid, with or without any trace of previous apoplectic extravasation, and who have suffered a first attack or a subsequent attack of fatal apoplexy, it is probable that the brain, from this cause, is rendered less impatient of injury from extravasation of blood. This position is exemplified in a remarkable manner in Case VI., of extravasation into the ventricles, and in Case X., of extravasation on the surface of the brain.

4thly. That a loaded state of the bloodvessels is sufficient to produce all the symptoms of sanguineous apoplexy, and to occasion death without extravasation. It is probable that, when this state of the vessels is found connected with only a small quantity of fluid in the membranes or ventricles, that death has been occasioned by simple sanguineous apoplexy. That convulsions in children arise in general from cerebral congestion, and are essentially cases of sanguineous apoplexy.

5thly. The conclusion is highly probable, that in very many instances of sudden or speedy death, which have been supposed to be occasioned by the presence of serous fluid, discovered on dissection in the membranes or ventricles, death is not attributable to this fluid, or to serous apoplexy; but the inference is much more reasonable, that these cases may be referred to simple sanguineous apoplexy, the fluid in the brain having nothing to do with the fatal event." 314.

We have laid a fair and sufficiently detailed account of the views of Dr. Sims before our readers. They occupy fifty pages of the Society's volume of Transactions, and must, therefore, be considered by himself as calculated to engage and to merit attention. He modestly informs us, in the prefatory observations, that his paper is intended to illustrate and confirm the opinions of others. This is not only modest, but true—the paper is calculated chiefly for this. There is in it little novelty of fact or of deduction—little which has not been said before—little, indeed, which cannot be found in the latest and best elementary treatises. The opinions enforced by Dr. Sims will be found expressed, with more or less distinctness, by Dr. Abercrombie, Cruveilhier, the Editor of this Journal, and by many others.

We were curious to see what was said by the latest systematic writers, and we referred to that admirable Dictionary of Dr. Copland, which more, perhaps, than any other work in medicine, embodies the opinions of the best authors and the best physicians. In the article upon the simple form of apoplexy, contained in the Dictionary to which we have alluded, we find the following remarks upon serous effusion.

"Serous effusion, in those cases in which it constitutes *even the chief lesion*, cannot be viewed in any other light than in that of a result of pre-existing disturbance of the circulation, depending, as will be more fully alluded to in the sequel, either upon imperfect vital tonicity or action of the vessels, or upon obstructed circulation, especially in the veins and sinuses of the organ, or even upon both. Another circumstance, well deserving of notice, and evincing that the serous effusion is of itself to be viewed as merely a part, and, indeed, no very important part, of the existing lesions, although the most demonstrable, is the fact also insisted on by Dr. Abercrombie, that the quantity of fluid effused bears no proportion to the degree of the apoplectic symptoms: for we find it in large quantity when the symptoms have been slight; in small quantity when they have been both strongly marked and long continued; and, finally, we find most extensive effusion in the head, where there have been no apoplectic symptoms at all. The inference, therefore, clearly deducible from the most faithfully observed facts, is, that the effusion is not the cause of the apoplectic seizure, but the consequence of that state of circulation on which the disease more immediately depends. Indeed, I am even of opinion that a considerable portion of the effusion takes place either immediately before death, or soon after life is extinct; and that several cases referred to serous effusion have not arisen from this cause, the quantity of serum having evidently not been greater than we have reason to believe naturally exists in the head, as necessary to the regularity of its functions, under the varying states of circulation, and of atmospheric pressure on the surface of the body, from which the unyielding bones of the cranium protect it." 81.

Dr. Copland expresses, in the preceding passages, not merely his own sentiments, but those of Abercrombie, of Cruveilhier, of the Editor of this

Journal, and of many others. As Dr. Sims remarks, his paper tends to give to those opinions additional strength. Facts in medicine are always useful, even when they prove a point already proven. For few truths are so established, as to derive no confirmation from additional investigations. But, in the present instance, some doubts existed in the minds of those unused to pathological researches; and erroneous notions on the subject of serous effusion are unquestionably entertained by ill-informed persons. Dr. Sims may, therefore, justly lay claim to the merit of illustrating and elucidating opinions, not yet so generally admitted as they should be.

### II. On Hypertrophy and Atrophy of the Brain. By John Sims, M.D.

This, the second paper contributed by Dr. Sims, occupies sixty-seven pages of the volume. Its principal portion is dedicated to the subject of hypertrophy, which is made by our author his cheval-de-bataille.

The Continental pathologists have lately drawn attention to the occurrence of what seems to be hypertrophy of the substance of the brain. The observations of British physicians have certainly not been numerous nor extensive. Dr. Copland, in his Dictionary of Practical Medicine, refers to the chief authorities upon the subject, and Dr. Sims appears to have followed in Dr. Copland's wake, quoting the same authors, and nearly the same passages. As in the preceding paper, on Serous Effusion, Dr. Sims appears to be actuated by the very praiseworthy intention of illustrating the opinions of others. The present paper may be viewed as an attempt to bring the views of those who have written on the subject of cerebral hypertrophy, more fully than has yet been done before the profession in this country. The article is too long; but we shall give a connected and sufficient account of it. As it does not contain much novelty, it does not appear to require an extended notice, yet its facts are not unworthy of attention.

Dr. Sims uses the term hypertrophy of the brain as referable to two states; one consisting in simple enlargement, the other where a change of texture in the cerebral substance exists. The following is the plan pursued by Dr. Sims in the construction of his paper. He proceeds, in the first place, to give a very short report of the opinions of a few authors who have written on the subject:—2. To relate the histories of several cases of hypertrophy which have fallen under his observation. 3. To attempt to ascertain the average weight of the brain of persons of all ages, from a very large number of cases in which the brain has been weighed. 4. To give short notices of a number of brains which exceeded the average weight, from various causes, but which scarcely come under the description of hypertrophy. 5. To relate the histories of several cases of atrophy of the brain, both general and partial. 6. In conclusion, to draw such inferences as the facts and observations, detailed in the paper, appear to warrant. The historical sketch we may omit, as our readers may either consult the paper itself, or, what is equally good, the article on Hypertrophy of the Brain, in Dr. Copland's Dictionary of Medicine. The latter has more of the merit of conciseness.

Dr. Sims details twelve cases of general hypertrophy of the brain, and three of partial hypertrophy of that organ.

CASE 1.—*Hypertrophy of the Brain—Death from Enteritis.*

A. B. æt. 16. She died from inflammation of the bowels. But she had a very large head, and was thought to have been hydrocephalic. She was subject to some kind of fits until she was seven years old: the catamenia appeared at fourteen; she had an enormous appetite; there was no paralysis, but a very great weakness of all her limbs; she complained much of giddiness, and had a bad memory; she had a difficulty in learning and performing the ordinary mechanical arts of her sex, as sewing, &c. During her last illness, there were no cerebral symptoms.

*Dissection.* The head appeared to be twice the ordinary size. The hands were very small. Ossification of the cranium complete. Brain: the arachnoid was much thickened by the deposition of slightly opaque lymph on the upper parts of both hemispheres. The cerebrum was very firm to the knife, and presented no unusual appearance except its enormous size: the quantity of brain appeared to be double that of a person of her age. The cerebellum did not partake of the enlargement. The ventricles contained only from two to three ounces of clear fluid. The cranium was not measured, nor the brain weighed. In this instance the brain was simply enlarged, without appreciable alteration of structure.

CASE 2.—*Hypertrophy of the Brain—Death from Extravasation of Blood into one of the Crura Cerebri.*

Martha Lock, æt. 40, had been in the Infirmary four years, when she fell to the care of Dr. Sims. She has for several years suffered at intervals severe paroxysms of pain referable to the pyloric end of the stomach and duodenum, accompanied with vomiting of large quantities of thin mucous and green bilious fluid, amounting to many pints during the paroxysm. The paroxysm is also attended with the most intense headache. Her sufferings have been sometimes so great as to deprive her of reason for a time, and at others to produce a state of coma. In one of the attacks she bit her tongue nearly half across, and more than once attempted to destroy herself in order to put an end to her agony.

June 8, 1832. For the last three days she has been suffering a paroxysm of very great severity, attended with violent convulsive agitation, and vomiting of dark green fluids, with constant purging. She had two fits of an epileptic character yesterday, and three this morning. She died suddenly about one, p.m.

"*Inspection, 24 Hours after Death.*—Head. A considerable quantity of blood escaped on dividing the skull. On removing the calvaria the dura mater was remarkably distended, and appeared protruding as from pressure within. The convolutions were much flattened, and their divisions almost obliterated. The medullary matter was throughout of a dead or flake-white colour; scarcely any red points appearing in the sections made of the bloodvessels ramifying through it. The substance of the brain appeared to be much increased in firmness and in bulk. The membranes were very dry, and the pia mater adhered very firmly to the surface of the brain. The ventricles were contracted in size, and contained no fluid. In the right crus cerebri, which was in some slight degree softened, there was a small clot of recently extravasated blood, the size of a pea. A small body, very much resembling an enlarged absorbent gland in any other part of the body, was found in the arachnoid near the basilar artery. Cerebellum natural. The brain was not weighed." 330.

The left ventricle of the heart was hypertrophied—the mucous membrane and pyloric valve of the stomach were thickened—and the mucous membrane of the upper part of the small intestines was in a highly vascular condition.

CASE 3. *Hypertrophy of the Brain—Convulsions—Stertor, &c.*

Joseph Trendal, æt. 22, admitted March 8th, 1833, shortly after having taken much mercury for syphilis. He was reported insane, but when visited he was quite rational, although unable to control the spasmodic motions of the muscles of his extremities, which were thrown about, and much distorted. Cupping, and other depleting measures, brought him into a quiet state; and he expressed himself as feeling much better. On the evening of the 9th he was suddenly seized with a fit resembling epilepsy, which soon however assumed a confirmed apoplectic character, attended with complete insensibility, stertorous breathing, and general convulsions. He died the following evening.

*Dissection, 43 hours after death.* Brain. The convolutions were much flattened, and closely pressed together. The bloodvessels were almost entirely empty. The membranes were quite dry, and the ventricles appeared to be almost obliterated, and would scarcely contain a dram. The structure of the brain was generally hard, firm, and of a pinkish hue. It weighed 3lbs. 9oz.

CASE 4.—*Hypertrophy of the Brain—Pulmonary Tubercles—Enlargement of the Heart—Sudden Death.*

James Tipton, æt. 40, admitted Dec. 31. He has cough, dyspnœa, purulent expectoration, and diarrhœa. He died rather suddenly, after rising from bed.

*Inspection, Jan. 8, fifteen hours after death.*—Head. There was a considerable quantity of fluid in the cavity of the arachnoid, and in the subarachnoid tissue. There were two large bladders on the superior margin of each hemisphere, which seemed to have broken down the filamentous tissue between the pia mater and arachnoid. The brain was remarkably turgid with blood, was firm, and cut like ground-rice pudding. Weight 3lbs. 7oz.

Thorax.—The lungs were studded throughout with tubercles, and there were small cavities in different parts of the lungs. Heart large, with considerable dilatation of right ventricle.

In the next case, the patient, aged 24, died of phthisis, but complained of headache, and was delirious for the four days preceding death. The brain weighed 3lbs. 5oz.; its convolutions were flattened, and there was ramollissement of the centre of the posterior lobes of the cerebellum.

In the sixth case, the patient, aged 70, while in her ordinary state of health, died suddenly. There was some fluid in the sub-arachnoid tissue—the brain weighed 2lbs. 15oz.—and the medullary substance of the brain was very white and cut like hardened custard. The heart was remarkably flaccid.

In the seventh case, the patient, æt. 29, had dropsy from diseased kidney. She died from diffuse inflammation of the face. The brain was very dense, its convolutions flattened, and its weight 3lbs. 2oz.

In Case 8, a woman, æt. 48, died in twenty-six hours of malignant cholera. The cerebral bloodvessels were full—the roof of the right ventricle adhered to the corpus striatum—the brain weighed 3lbs. 5oz., and, when cut into, resembled coagulated albumen, with a quantity of plaster of Paris mixed with it.

*Case* 9. "Dec. 14, 1832, I was requested by Mr. Jorden, of Belgrave Street, to accompany him to examine the body of Caroline Bays, a child, aged eleven months. I received from him the following particulars of the child's history. Her head was observed to increase in size in an unusual degree about six weeks after birth: the general health was good until about three days ago, when she became feverish, with rapid pulse and constipated bowels. At this time the head was much enlarged, the forehead being remarkably prominent; the fontanels enlarged, firm, and tense to the touch; superficial veins enlarged; the eyes were generally fixed in a downward direction, more especially the left. The child was constantly moaning, and rolling its head from side to side and tossing its hands about. Convulsive twitchings latterly came on. The mother has had four children, all of whom died either a few days or weeks after birth. Leeches, cold lotions, and purgatives were used without effect.

*Inspection, eight hours after death.*—Head. The fontanels were large, sunk, and flaccid: cranium of rather unusual thickness. The circumference of the cranium, after removing the integuments, at the largest axis measured seventeen inches and a half, and from one meatus auditorius internus, over the vertex to the other, twelve inches. The membranes and substance of the brain were bloodless. The brain was very much enlarged, and its consistence rather softer than usual. The ventricles contained about one ounce of fluid." 341.

In Case 10, the patient, ten years old, died of cholera, having been previously quite healthy and intelligent. The convolutions of the brain were flattened—the grey matter was soft, the medullary matter very white, the substance of the brain cut like blanc-mange, or thick custard, and its weight was 2lbs. 14oz.

In Case 11, a child, æt. 3, died of pneumonia after hooping-cough. She was insensible, with coma and occasional convulsions for the last 24 hours. On inspection, the signs of hypertrophy, with excessive fulness of all the veins and sinuses of the brain. The latter organ weighed 2lbs. 15oz.

In Case 12, a girl died of malignant cholera. The brain was enormously enlarged, weighing 3lbs. 12oz.

Such are the cases of general hypertrophy of the brain, brought forward by Dr. Sims. The majority we have greatly abridged, but we have inserted the leading and most important particulars. Three cases of "partial hypertrophy" follow; Dr. Sims observing that it is frequently found that parts of the brain are enlarged beyond their ordinary size, either originally, or in after-periods of life.

CASE 13. *Hypertrophy of one Hemisphere—Old Apoplectic Cysts.*

"Esther Walters, æt. 60. She is reported to have had a paralytic attack about two years ago, from which she was much relieved. She continued to move about until within ten days of her admission. Signs of arachnitis came on with coma, and she died June 21st.

*Inspection.*—Head. The left hemisphere occupied about two-thirds of the space allotted to the cerebrum. The left corpus striatum was enlarged to twice its usual size: the right corpus striatum was diminished in size. There were two apoplectic cysts in the left hemisphere and one in the right, of different dates." 347.

In Case 14, that of a lunatic for twenty years with lucid intervals, the skull was very hard—much arachnoid effusion—the corpora striata unusually approximated—right corpus striatum twice its ordinary size—left slightly enlarged—left thalamus much enlarged—tuber annulare half as large again as usual—crura cerebri enlarged.

In Case 15, that of a pot-boy, æt. 29, who died of delirium tremens, the posterior lobes of the cerebrum are said to have been greatly enlarged. The brain weighed 3lbs. 8oz.

We are now prepared to listen to the conclusions of our author. We have seen the data, at all events the chief of them, on which his opinions are principally founded, by which he wishes them, we presume, to be determined. Let us then listen to Dr. Sims.

"The state of the brain" he says, "which I have described admits of division into two kinds; one, a state of mere enlargement or addition of particles, without any appreciable or apparent difference from the ordinary state of the organ; this appears to be connate, or connected with rickets, manifesting itself very early, and attaining in some instance a very great magnitude. In the other and most important form of hypertrophy, the weight of the brain is sometimes very materially increased; but in this case it is not of so much value as the bloodless state of the brain, its freedom from serous effusion, its very white and albuminous texture, and the flattening of the convolutions, &c., which I have so often noted.

If we could ascertain with some degree of accuracy the average weight of the healthy brain at various periods of life, we should then be able to estimate more nearly the extent of hypertrophy, so far as the circumstance of weight is concerned; but almost all the attempts that have hitherto been made have been ineffectual and liable to error. The method to fix the weight of the brain on a scale, comparing it with the weight of the body, does not remove the difficulty, for some very thin subjects, exhausted by wasting diseases, have brains above the average weight, and it is well known that some of the heaviest bodies possess very small brains.

Haller states that in one child, a boy of six years old, he found the brain to weigh 2lbs. 28½ drams, the weight of the body being 50lbs. or about $\frac{1}{22}$:—in other instances he found the weight of the brain to be $\frac{1}{25}$, and $\frac{1}{30}$ the weight of the body. He further observes, 'Homini cerebrum aliquando libram cum semisse in adulto homine non superavit: alias fuit trium l., et trium l., atque 8 unciarum cum ¼, et quatuor librarum; quatuor porro et unciarum trium; quatuor et unciarum tantumdem, quatuor ad quinque; quinque denique et ultra.'*

Soemmerring, in the section 'Pondus Cerebri,' remarks, 'Cerebrum et cerebellum, resecta medulla spinali statim pone nervum lingualem medium, pondo sunt librarum duarum, ad tres libras, sunt enim alia cerebra pondere librarum duarum, et unciarum quinque cum dimidia, alia librarum trium et unciarum trium cum tribus quartis.'† His 'Tabula Baseos Encephali' is taken from a child three years old.

The Wenzels, in their great work on the structure of the brain, state, 'Pondus encephali humani quale id de quinto vitæ anno ad summam usque hominis senectutem plerumque invenitur, pondus viginti quatuor millium granorum non superat.' Again, 'Totius cerebri pondus interviginti et viginti-duo millia gra-

---

* Elementa Physiologiæ, Tom. IV., p. 10.

† De. Corp. Hum. fabrica, Tom. IV. p. 38.

norum plerumque variat.'* They give a table of the weight of nineteen brains of various ages, from a fœtus of five months up to old age, 88 years.

Sir William Hamilton has taken great pains to ascertain the weight of the brain, and concludes, 'that the adult male encephalos is heavier than the female; the former nearly averaging, in the Scot's head, 3lbs. 8oz. troy, the latter 3lbs. 4oz., the difference 4oz. In the male, about one brain in seven is found above 4lbs. troy; in the female hardly one in a hundred.' Sir William founds this and other conclusions, 'on an induction drawn from above *sixty human brains,*—from nearly *three hundred human skulls,* of determined sex, the capacity of which, by a method I devised, was taken in sand, and *the original weight of the brain* thus recovered.'† I think the weight of the brain cannot be fixed by ascertaining the capacity of the skull, by any means however accurate; it is open to a source of fallacy, which appears obvious on inspecting the last column of Table I." 352.

Dr. Sims has endeavoured to ascertain the weight of the brain in a great number of dissections of persons of all ages and both sexes, who died of diseases of almost every description, whether cerebral or otherwise: in some the brain was healthy, in others diseased in different degrees and forms. He presents a table taken from 253 dissections of the brain. The weight used is avoirdupois, and the weight of the whole encephalon is taken, the dura mater being removed. The sex, age, disease producing death, and weight of the brain, are the things specified; and a notice is added of whatever might influence the weight.

The various Tables appended by our author are too voluminous for insertion here, and we must therefore confine ourselves to the results. Such of our readers as would have all the data before them must refer to the volume of the Society's Transactions. Prior, however, to stating the results, we find we must advert to atrophy of the brain.

In persons of advanced life, the brain is frequently noticed to undergo a marked diminution in magnitude. In young persons, too, who have suffered from long-continued wasting diseases, the brain has suffered a general wasting in all its parts, both grey and white.

" The marks by which this state of wasting may be known are, a particular collapse or shrinking together of the convolutions when the horizontal section is made, so as to cause the white matter to appear depressed, and not to present the even surface observed in a firm and healthy brain;—a quantity of serous fluid may be squeezed out of the interstitial tissue of the brain;—a stringiness of the substance, arising from the absorption of the pulpy matter, and the probable thickening of the filamentous tissue;‡ flaccidity of the brain in some instances; a dilated state of the blood-vessels, whether empty or filled with blood; the presence of serous fluids in the ventricles or membranes, and hypertrophy of the bones of the skull.

In partial hypertrophy we sometimes observe one hemisphere of the cerebrum to be very much less than its fellow; one lobe of the cerebellum also to be diminished in comparison with the other; sometimes parts of the grey matter covering the convolutions are dwindled. Very frequent instances of partial atrophy are met with in the corpora striata and in the thalami. The natural

---

* J. et C. Wenzel, De Penitiori Structura Cerebri, p. 267.

† The Anatomy of the Brain, &c., by Dr. Monro, 1831, p. 4.

‡ Dr. Baillie has remarked that the brain is sometimes tougher and more elastic than usual, and that under these circumstances the ventricles were enlarged in size and full of water.—*Morbid Anatomy,* p. 434.

rounded form of the corpus striatum is rendered much flatter, and there are often furrows in it; the extent of its surface which appears in the ventricle is much contracted, and on cutting into it, the grey matter appears loose, and holes are frequently observed. The thalami are sometimes much wasted, the contiguous sides are widely separated, and instead of the perpendicular line of the lateral face of these bodies, it appears scooped or hollowed out; their rounded or upper surface is either flat or hollowed, and the ventricular margin presents a ridge instead of the usual convexity. One of the thalami, or one of the corpora striata, is sometimes atrophied, whilst its fellow retains its normal state, or is hypertrophied." 366.

Dr. Sims thinks that the blunted feelings, loss of memory, impaired movements, &c. of old persons may be accounted symptoms of cerebral atrophy. He even goes so far as to suppose that the muscular weakness and general emaciation of phthisis may in some degree depend on wasting of the brain. This is, as Trim says of my Uncle Toby, somewhat hobby-horsical. Blunted feelings, impaired movements, and so forth, are the common accompaniments of old age; no octogenarian but exhibits one or all more or less. But even Dr. Sims does not, we suppose, contend that all old people suffer from atrophy of the brain. Nay, he cannot, because he is bound to provide for hypertrophy, which is also discovered to be frequent. In phthisis, too, emaciation always occurs, but we do not conceive that, common as atrophy may be, it is invariable. Indeed, some persons, considering the great expectoration, the night-sweats, the diarrhœa, might look on atrophy as caused by the emaciation or by what occasions it—not on the emaciation as resulting from the atrophy. Yet it is pleasant to see with what strides pathological knowledge marches on. It was but yesterday that people stared when cerebral hypertrophy or atrophy was talked of—to-day we find them both as plenty as blackberries. But of this anon.

The chasm, continues Dr. Sims, occasioned by the atrophied state of the brain, we observe to be sometimes filled up by serous fluid, and by deposits of bone on the skull: and the opinion is highly probable, that this excess of fluid and of bone is placed there to supply the deficiency. If the brain wastes, it is quite clear that something must be placed in the unyielding cranial cavity instead of it. It may be hard in some cases to determine whether the atrophy of the brain gives rise to the serous effusion and the osseous depositions, or whether the latter have been the causes of the atrophy.

Dr. Sims presents the details of four cases, and notices of twelve others, in which more or less atrophy of the brain existed. We will extract the first and the third cases.

*Case.* E. Burgess, æt. 70. About four months ago he was seized with apoplexy, attended with hemiplegia of right side, and loss of speech. Since the attack he has never recovered the use of the paralysed side, but he has had occasional variations of sensation and motion. He soon shewed signs of childishness, which increased nearly to fatuity. He made frequent attempts to speak, and sometimes he succeeded in uttering a word or two.

*Inspection, Jan.* 13, 1835, *ten hours after death.* Head.—The cranium was thickened by a deposition on the inside of the os frontis, so as to diminish the capacity of the frontal region of the cavity: the corresponding parts of the anterior lobes of the cerebrum were diminished in size and flattened. There was considerable opacity of the membranes, and effusion into the ventricles and membranes. The right corpus striatum was flaccid and shrivelled, and of a reddish-brown colour. Both the thalami were atrophied to about half their natural size. The remains of an apoplectic cyst were found in the left corpus striatum. Weight of the brain, 2lbs. 4oz.

Dr. Sims thinks, and probably with justice, that the wasting of the brain began at the time of the apoplectic attack, and that as it advanced the fatuity became established.

*Case.* Jane Hackett, æt. 41, admitted in the last stage of exhaustion from pulmonary tubercles. She had slight diarrhœa, which was easily checked; and was greatly emaciated. She had no head symptoms.

*Inspection, forty-four hours after death, May* 12. Head.—Cranium hypertrophied, in front especially, but also over the whole calvaria. Three ounces of fluid were found in the ventricles, and a very large quantity in the membranes, in some parts of a dusky colour. The vessels of the membranes were dilated, and those of the brain highly congested. The substance was soft from interstitial fluid, otherwise it was natural. Weight, 2lbs. 14oz.

The lungs were extensively tuberculated.

We think we may omit the notices of instances of atrophy of the brain. In some cases there were symptoms referable to cerebral affection, in others there were none. Examples enough of hypertrophy and of atrophy have been presented to enable us to follow our laborious author in his inferences and conclusions. Those conclusions we must present entire; they cannot be abridged.

"1. From the short historical sketch it appears that pathological writers have hitherto had a very imperfect knowledge of the phenomena connected with hypertrophy of the brain. M. Laennec did not observe the peculiar changes of structure which the brain presents in this affection. Scoutetten notices the greater consistence of the brain. Dance is the first to take much notice of the change of texture, though in his definition he limits the change to a mere addition of molecules. Portal, Otto, and Andral, have added several facts. British pathologists afford but faint indications of an acquaintance with the affection.

2. That cases of hypertrophy of the brain are met with where no change of texture can be discovered, and the enormous size of these brains arises from the mere addition of similar particles.

3. That cases of hypertrophy occur, in which, added to the increased size of the brain, there is a change in the texture of the brain, which has been described as resembling boiled albumen, ground-rice pudding, cream cheese, &c.; a flattening of the convolutions; little or no blood or serous fluid in the vessels, cavities, or membranes of the brain. That this state of the brain is of a more acute character, and is probably produced more or less rapidly by any causes that excite the brain or its blood-vessels, or that increase general or partial nutrition.

4. That hypertrophy is allied to or connected with apoplectic seizures, either as a precursor, a concomitant, or a cause: that in this state of the brain, simple sanguineous apoplexy may be more readily induced, or life may be destroyed by a very small clot of extravasated blood.

I have no doubt that in the dissection of apoplectic brains, many cases of hypertrophy have been entirely overlooked, and the brain has been reported as normal; now, believing that I am correct on this point, I take the liberty of impressing on the minds of medical practitioners the further investigation of this subject, in consequence of its obvious bearing on the practical treatment of apoplexy and other diseases of the brain.

5. That extensive disorganization of the heart and lungs may impede the return of blood from the brain, or so obstruct its circulation as probably to occasion hypertrophy, or enlargement of the brain.

6. It is probable, that in cases of sudden death which on dissection have been attributed to a flaccid state of the heart, angina pectoris, spasm of the heart, &c. hypertrophy of the brain, causing simple sanguineous apoplexy, though unnoticed, may have been the sole cause of death.

7. That in brains that are hypertrophied, both children and adults are sometimes destroyed by active inflammation of the brain, terminating in ramollissement: and also that this affection is frequently connected with the more slow form of ramollissement in adults, whether arising from inflammation or not.

8. That in some children who, from the size of the head, are suspected to be suffering under hydrocephalus internus, or the disease terminating in the deposition of fluid in the ventricles, it is highly probable that the brain is in a state of hypertrophy.

9. That the brain is sometimes affected partially by hypertrophy, either of one hemisphere, one lobe, or of the corpora striata or thalami.

10. That hypertrophy is confined to the cerebrum; the cerebellum is not so affected.

11. From an extensive series of observations, it appears that the average weight of the brain goes on increasing from 1 year old to 20; between 20 and 30 there is a slight decrease in the average; that afterwards it increases, and arrives at the maximum between 40 and 50; that after 50 to old age, the brain gradually decreases in weight.

12. That the brain is sometimes unusually large, not amounting to hypertrophy, in persons dying of various diseases, especially in extensive pneumonia, and other diseases of the lungs and heart; and in these cases the brain is generally very much loaded with blood.

13. That the brain in the advanced periods of life, and in some diseases, is diminished in volume or atrophied, either generally or partially; and that there are certain marks observed in dissection, by which this state of the brain may be known. It is also probable that there are symptoms occurring during life, indicating atrophy of the brain.

14. That in phthisis pulmonalis, diseases of the stomach, and other emaciating disorders, the brain also sometimes undergoes a process of wasting.

15. That in cases of atrophy of the brain, the place previously occupied by cerebral substance is supplied by serous fluid, or by deposition of bone; and this deposit of bone frequently takes place on the inner surface of the cranium, sometimes in the diploe.

16. That the cerebellum is subject to atrophy, as well as the cerebrum." 380.

On reverting to his facts, and casting an attentive glance at his conclusions, it is impossible, and it would be wrong to attempt, to deny to Dr. Sims the merits of industry and ingenuity. It is obvious that he has toiled more than any pathologist in this country, to improve our acquaintance with the nature of hypertrophy and atrophy of the brain. How he has succeeded is another question, and will probably be determined in different ways by different inquirers. For our own parts, we must say that we think he has thrown *some* degree of light upon the curious affections he has investigated, though we cannot go so far as he goes in the inferences that he draws from his observations. It is singular, indeed, that what was generally considered, to this moment, an obscure, a rare, and a dubious alteration, should now, if the observations of Dr. Sims be correct, appear in the light of an every-day occurrence. It was but yesterday that the mention of the name—hypertrophy of the brain, was certain to excite an incredulous smile; now we have cases plenty as blackberries, and, did we accede to the views of Dr. Sims, it is continually occurring and continually overlooked.

We know not what to say: on the one hand, the detailed cases of hypertrophy appear to shew that such a thing is—on the other, reasoning raises plausible objections to its history. From the account of Dr. Sims, hypertrophy of the brain would seem to occur at all periods of life. In advanced age, we do not understand how it is possible for the cranium to enlarge. So soon as several of the sutures are united, augmentation of the bulk of the cranial cavity would appear to be impossible. The only increase of its size can be effected by the partial absorption of the cranial bones. An actual increase of bulk of the cerebrum would appear unattainable in middle and in after-life. To this we presume it will be answered, that though the periphery of the brain may not enlarge, yet fresh molecular deposition may occur at the expense of the removeable contents of the cranium—the blood and serous fluid. In proof of this, the advocates of hypertrophy would refer to the flattened convolutions, the bloodless condition of the cerebrum, the paucity of serum in its ventricles, and on its surface. Yet again the cautious pathologist hesitates. We know how various the conditions of the brain are, when apparently its functions were performed aright. There is no certain standard by which to determine what is, or what is not, a natural condition of brain. Any marked deviation is discernible, and may be allowed—but any deviation that is not marked, is too vague to furnish to the philosophical inquirer unquestionable or satisfactory data. How, then, are we to judge of hypertrophy? By the common evidence of common sense? That is very difficult. By the standard of averages? That is not yet clear. Averages presuppose extensive observations. Great as has been the industry of Dr. Sims, we cannot consider his observations sufficiently extensive.

If we turn to symptoms, the same uncertainty surrounds us. One patient dies with marked cerebral symptoms—hypertrophy is found upon dissection, and cause and effect seem plainly brought before us. But another patient dies, who never has exhibited cerebral symptoms at all, and again we find hypertrophy. What are we to say? The cautious reasoner is staggered, and he pauses for further information.

Dr. Sims, indeed, is sanguine. He does not appear to feel the difficulties we have pointed out. When symptoms existed, he is satisfied with the explanation that hypertrophy presents—when no symptoms were present, he is, or seems to be, satisfied still. For our own parts, we repeat, we know not what to say. Mr. Boswell used to observe to Dr. Johnson—"What won't fill a quart-pot will fill a pint." We should be sorry to arrogate bulk of any description to ourselves, but really our author on this occasion, represents the pint-pot, and we are swollen to the dropsical dimensions of the quart. The evidence does not half fill us.

That the brain wastes, especially in old age, is probable in itself, and, what is better, is demonstrable. But, as the brain wastes, something in the cranial cavity must supply its place, and that something is generally serous effusion. In many instances, it must be very doubtful which of these organic lesions is cause, and which is merely consequence. Serous effusion must occasion diminution of the brain, and diminution of the brain is very likely to give rise to serous effusion. However this may be, we cannot feel surprise at wasting of the cerebral substance in old age. It obeys only the law to which all the other tissues submit. Yet, even in old age, atrophy is but one of many changes of the cerebral mass. Its arteries are become opaque, obliterated, or dilated—its venous system is enlarged, in a state of congestion, and its current dull and sluggish—the cellular tissue below the arachnoid is the seat of serous effusion—and, finally, the brain is wasted—and its structure probably altered. Can we single out one of these many changes, and on it alone lay the blame of the intellectual decay of the octogenarian? We might as well pick out some particular alteration in some other tissue, and attribute to it the declining performance of that tissue's functions. In old age, all the textures and all the systems are deteriorated, each in its turn and in its way; and, as every part is composed of many subordinate portions, it is the sum of their alterations that constitutes the infirmities of declining years.

We cannot go so far as Dr. Sims in many of his inferences, or rather suppositions. Having found hypertrophy in persons who were intellectually sound, and who lived and died without a cerebral symptom, we cannot see with what confidence our author can suppose that many instances of sudden death may be found to depend on such hypertrophy. Such suppositions are obviously hypothetical in a high degree. Yet it would be expecting too much from human nature, to call upon a man to pursue long and closely a particular investigation, and to give to its details no more than they deserve.

### III. On the Cure of Ramollissement of the Brain. By J. Sims, M.D.

The objects of our author in this paper cannot be so well or so clearly stated as in his own words.

"Two varieties (he commences by observing) are principally observed on dissection, in the early stages of ramollissement of the brain: one, of a red colour, chiefly taking place in the gray matter covering the convolutions, or in that of the corpora striata, &c. and not unfrequently in the white matter, when connected with apoplectic extravasation of blood; the other, of a white colour, or sometimes of a pale yellow colour, occupying the white matter of the brain. This frequently occurs in the central parts of the brain of children, and of adults who die of the inflammatory disease termed hydrocephalus acutus: it is also frequently met with in the brains of old people connected with diseased bloodvessels, and other morbid appearances, and is then most probably not an inflammatory affection.

The colour of the red ramollissement appears to arise from the capillary vessels, carrying red blood, being dilated in the softened spots, from extravasation, or from transudation through the coats of the vessels, or probably from all these circumstances taken together.

The colour of the white ramollissement is probably occasioned by the softening taking place in the intensely white part, where no red capillaries are seen, as the corpus callosum and the fornix, or in the white matter where red vessels are seen sparingly, it may take place in small medullary patches, at a little distance from these vessels, so as not to endanger their laceration; or in cases of diseased arteries, the red vessels in patches of the white matter have become ossified, cartilaginous, or obliterated; or the colour may be influenced by the admixture of lymph or puriform fluid with the softened parts of the white matter.

In examining the brains of persons who have in a former period suffered apoplexy, paralysis, vertigo, loss of feeling or motion, we find generally some or other of the following appearances: cicatrices; cysts, containing less or more fluid; also an ochre or fawn-coloured matter, the evidence of former extravasation; small holes of various sizes, lined by a thin, transparent membrane, and containing serous fluid; atrophy of the gray and white matter; small spherical lumps of coagulated blood; serous fluid in the membranes, the ventricles, and the substance of the brain.

I am inclined to believe the atrophy of the gray matter, whether on the surface or in the central parts of the brain, when a fawn-coloured deposit is connected with the wasted spots, and the numerous small holes in the white matter with yellow deposit, as evidence of the arrest or cure of red ramollissement: and the clean-cut cavities in the white matter, and sometimes in the gray matter attached to it; and very often in the gray matter of the corpora striata; the numerous small holes lined by a pale membrane, vascular or not, and containing serous fluid; also the new bread, or porous cheese-appearance, and the small, harder lumps in the white matter, and the general hardening of it, as evidence of the arrest or cure of white ramollissement. In the following pages I shall endeavour to illustrate, and *I trust* to prove, the truth of this position, by several dissections of the brains of persons in whom these appearances were found." 383.

The paper occupies thirty-seven pages of the volume. It contains twelve cases. Our author, after the preliminary observations we have quoted, revives the opinions of pathologists on the subject of ramollissement and its cure. M. Cruveilhier appears to be the one who has been most explicit, and indeed most sanguine, in his ideas of the mode of reparation in this malady. M. Cruveilhier's sentiments may be stated.

The mode of cicatrization, he says, of ramollissement of the white substance, is occasionally exhibited in the formation of cellules, infiltrated with a sort of clear bouillie, like size; while the mode of cicatrization of ramollissement of the grey substance consists in cavities with a yellowish scar. Such is M. Cruveilhier's account of the cure of ramollissement, of the white matter and the grey. He enters more in detail into the subject, but the preceding quotation is sufficient to explain his general affirmations.

Dr. Sims, indeed, goes farther than M. Cruveilhier, or, indeed, than any one else. Whilst Andral and others have doubted if such a thing as the cure of ramollissement occurs, Dr. Sims asserts unhesitatingly that it is frequent. A pregnant instance this of the differences of doctors! Yet exact investigations, such as those of pathology, would seem, â priori, to admit of few disputes of this sort. But to proceed. After criticising some cases related by Dr. Bright and M. Scoutetten, our author brings forward his own cases. They are, as we have already stated, twelve in number.

CASE 1.—*Recent Ramollissement—rouge on the Surface: Traces of Ramollissement cured in the Central Parts.*

Mary Green, æt. 60, admitted with symptoms of gastro-enteritis, of which she died. Two days before death slight delirium came on, succeeded by coma, and apparently attended with loss of sight.

"*Inspection, May 9th,* 1835, *twenty-five Hours after Death.* Head. High congestion of the veins of the surface, which were beautifully tortuous. The blood-vessels of the brain were loaded throughout. In the cavity of the arachnoid there was extravasation, and coagulum on some parts of the brain, in small thin layers. It might appear doubtful whether this was a post-mortem appearance, or one which occurred in the last moments of existence; but it is highly probable that it was connected with some spots of red ramollissement which appeared on the inferior part of both posterior lobes of the cerebrum. This was small in extent, and little more than a third or a half the depth of the gray matter, which was red from torn vessels and slight extravasation, so as to present the appearance of being worm-eaten. The arachnoid and pia mater adhered to the brain partially at these points. There was a fawn-coloured hole, the size of a pea, in the right thalamus. In the gray matter of the left corpus striatum there was a small cavity, with fawn-coloured lining, and around it were several small holes, without fawn-coloured deposit. There did not appear to be any holes in the medullary matter. Effusion of serous fluid in the sub-arachnoid tissue, of a gray colour. Four ounces of fluid in the ventricles, of old standing. Weight, 2 lbs. 4 ozs." 393.

There were several cicatrices of old cavities in the upper part of the lungs —globular hypertrophy of the left ventricle of the heart, with dilatation of the right chambers—ulcerations, &c. in the intestines. The following are our author's remarks.

"In this patient's brain, there appear to be traces of ramollissement in different stages of cure. In the softened spots on the surface of the posterior lobes of the hemispheres, absorption had taken place, and adhesion between the pia mater, arachnoid, and brain. The several small holes without discoloration around the larger cavity, lined by a fawn-coloured membrane in the grey matter of the left corpus striatum, appear to me to attest the pre-existence of a softened state of the brain surrounding an extravasated clot, and that these cavities are the traces of its arrest and cure." 393.

The second case detailed by the Doctor is interesting, independently of his ideas on the cure of ramollissement. We accordingly subjoin it, curtailing the particulars.

Case 2.—*Several Attacks of Paralysis—Ramollissement—Holes in the Brain—Spherical Lumps of Coagulum.*

Mrs. A. S. æt. 50, having suffered much anxiety and fatigue, and having experienced nervous uneasy feelings in the head, lost, on the 22d Nov. 1831, the due control over the muscles of the voice. The pulse was 100, sharp and contracted. On Dec. 1, she was suddenly seized with great nervous excitement and alarm, and experienced a change of sensation in her left hand, and a dragging of her left leg. These symptoms soon improved, and she gradually recovered nearly her former state; yet her nervous agitation occasionally appeared, and at one time it bordered on incoherence. On the 1st April, 1832, she had another attack. On the 3d, her articulation was very indistinct; left eye protruding; left angle of the mouth drawn down; diminished power of moving the left upper extremity, particularly the fore-arm, with very feeble power of grasping; no loss of sensation in the limb; no impediment in walking. Pulse small, sharp, and quick; heart beat with much labour over a large surface. Her general manner was altered, and her understanding and feelings appeared to have sustained a shock. She recovered from some of her symptoms, but the paralysis of the left arm remained. During the succeeding eighteen months, she suffered several other attacks, of more or less severity. Her intellect became enfeebled, and her feelings blunted. She declined in every way, and, in the beginning of 1834, she died.

*Inspection, the Day after Death.* Head. Skull hard, with a thick os frontis. The arachnoid and velum interpositum were opaque, and of a light slate colour: there was some fluid between the membranes, and from four to six ounces in the ventricles. There was an old fawn-coloured deposit in the left hemisphere of the cerebrum: the thalamus of the right side was softened. The blood-vessels were thickened with white deposit. Many small holes were observed, appearing like worm-eaten cavities, in both hemispheres of the cerebrum; some were also found in the cerebellum; they were of various sizes, some spherical, and others of an irregular shape; some were quite clean, and not at all discoloured, especially in the white matter; others were lined by a light fawn-coloured membrane. Several small black spherical bodies were observed, much resembling melanotic matter, in two or three places: they were afterwards found to be portions of coagulated blood inclosed in a membrane; the surrounding white matter was not tinged by the blood. The substance of the cerebrum, with the exception of the right thalamus, was harder than usual.

Thorax.—Hypertrophy of left ventricle of the heart. Other viscera examined, natural.

Dr. Sims' explanation of the preceding case may be omitted. It is more ingenious than satisfactory—probable, perhaps, but difficult of proof. He says, for example, that "the various holes found in different parts of the cerebrum and cerebellum, the fluid effused, and the hardening of the cerebrum, are traces of previous disease in the organ, which I take to have been ramollissement without extravasation, excepting in the holes where yellow matter was found." This may or may not be the case, but as such conjectures prove nothing, we may at present pass them by.

The next case we shall offer (the sixth of Dr. Sims), is brought forward as an instance of cure of ramollissement of the gray matter.

CASE 3.—Richard Bailey, æt. 53, brought to the workhouse April 10th, 1835, in a state of diarrhœa and exhaustion. On the 22d he died.

*Dissection.* The arachnoid lining the dura mater laterally, and throughout nearly the whole of the base of the skull, was generally, and more particularly in patches, discoloured by a granular deposit of minute rusty-coloured points, evidently the traces of effused blood between the dura mater and the arachnoid. The arachnoid on the right parietal region was separated from the dura mater. Several of the convolutions at the surface of the base of the posterior lobes were in various degrees absorbed, and the fawn-coloured deposit placed there; in one or two places, the whole of the gray matter was removed and the white matter exposed, in others an external thin layer only, the arachnoid covering these places was entire. From 8 to 10 ozs. of serum were effused between the membranes and into the ventricles. The brain was atrophied. The vessels were but little loaded, and had no appearance of disease. Weight, 2 lbs. 7 ozs.

There was fluid in both pleural cavities and in the pericardium—tubercles, and cavities, and pneumonia, existed in the lungs.

Dr. Sims looks on the absorption of the gray matter and the fawn-coloured deposit as ample evidence of the previous existence of red ramollissement with slight extravasation.

The heading of the tenth case is illustrative of the many lesions found in the kind of cases that fall in the workhouse under the inspection of Dr. Sims. His sexagenarian patients have probably, many of them, hardly one organ sound. The lesions of old age are so numerous and so general, that it is difficult to determine the contribution of each towards the mental and bodily decay. But we spoke of the heading of this case. It is styled one of—apoplexy—hemiplegia—attacks of angina pectoris—ramollissement of gray matter—traces of ramollissement in central parts—hypertrophy of the heart—ossification of the coronary artery. With this enumeration we shall rest content, and proceed at once to the conclusions of our author. He thus advances them.

"1. In some of the cases the account of symptoms is necessarily very limited, but this circumstance I do not consider of much importance, as I am desirous of placing the subject on its true basis,—the anatomy of the brain: in other cases, however, we have a sufficiently copious detail of symptoms which indicated the nature of the disease, and the progressive improvement in some of the paralytic symptoms corresponded with the traces of the cure or arrest of ramollissement observed in the brain. The cases, No. II., Mrs. A. S. and No. X., Thomas Wood, are examples in illustration.

2. The traces of the cure of ramollissement of the gray matter are, absorption of one or more layers of this substance on the convolutions, and adhesion of the pia mater to the part; holes in the gray matter of the corpora striata and other central parts, together with atrophy and flattening. When transudation from the blood-vessels, or extravasation, has taken place, constituting red ramollissement in the gray matter, a permanent fawn colour of the atrophied convolutions, and of the small holes in the other parts, is observed. The slightest form of this softening of the gray matter is noticed in the case of purpura hæmorrhagica; in others we have one or more layers removed, or the entire gray matter, leaving the white matter of the hemisphere visible. We sometimes see merely small holes in the corpora striata; at others, cavities of various sizes and forms, with a marked wasting of these bodies.

3. The traces of the cure of ramollissement in the white matter are, the numerous clean or scooped out holes containing a limpid fluid, some of which are observed to be lined by a fine transparent membrane; others appear as if worm-eaten. These holes are of various sizes and forms, from minute points to the magnitude of a bean; the porous cheese or new bread appearance; the hardened state of the white matter generally in these brains, and particularly in the parts contiguous to the holes; the granular state of the white matter indicating cicatrices; the hardened state of the corpus callosum, fornix, &c., found in the brains of children and young persons, with fluid in the ventricles, probably the consequence of previous inflammatory ramollissement at an earlier period of life. When there is observed the fawn-coloured deposit in these holes of the white matter, they are traces of red ramollissement of the white matter, or probably, in some instances, of what has been sometimes termed capillary apoplexy.

4. I have perhaps to assume in this argument that ramollissement is *by some means or other stopped in its progress;* and, if this be granted, the preceding detail of the morbid appearances will readily fill up the several steps of the process of cure: the absorption of the softened parts; the adhesion of the pia mater on the surface; the secretion of fluid into the holes; the granular state of the white matter, probably from cicatrization; the hardening of the white matter, probably from effusion of lymph; and the fawn-coloured traces of previous transudation and extravasation. And I think this assumption ought to be granted, from the improvement in the symptoms of some of the patients, corresponding with the state of the brain after death; and because it is consistent with all analogy in the powers by which nature arrests or cures disorganization in other parts of the body.

5. There are some points connected with the subject, as the spherical spots of coagulated blood, the extravasated layer on the arachnoid, the fawn-coloured trace of extravasation between the arachnoid and the dura mater, and the connection of the traces of previous ramollissement with apoplectic clots: these are subjects well worthy of attention, but I have sufficiently adverted to them in the remarks on the respective cases in which they occurred.

6. The preceding facts and observations are, I believe, sufficient to attest the cure of ramollissement of the brain, and to set the question at rest on the solid basis of pathological anatomy." 416.

It is difficult to know exactly what to say on the preceding observations. That Dr. Sims is satisfied is clear—that he mixes with his facts a large share of speculation is quite obvious—but that those facts are deserving of consideration cannot in fairness be denied. The cure of the red ramollissement attendant on extravasation is unquestionable. There is scarcely a hospital pupil of two or three years standing who is not perfectly familiar with the fact, that in the brains of persons who have had hemiplegiac attacks, and sometimes when no such history can be obtained, the cerebral mass displays here and there the marks of former extravasations with their attendant softening, in a condition more or less advanced towards cure. But we shall not pretend to determine if our author's general statement be correct or not—we shall not pretend to pronounce on the question whether he is right or wrong in his descriptions of the cure of the red and the white ramollissement.

One circumstance has struck us, and must make an impression on the minds of all—that the examinations so pursued by Dr. Sims were all in the bodies of old persons. The youngest of the individuals, who form the subjects of the cadaveric researches of our author, was 53 years old;—the majority were from sixty to seventy years of age. At that period of life, the lesions of the organs, more particularly of the brain, are comparatively seldom simple. The great fault is usually in the circulating system. The vessels are more or less inelastic, perhaps actually diseased—the left ventricle of the heart is probably hypertrophied—the various organs are more or less altered in their several ways—sub-arachnoid effusion is found in the brain—the texture of that organ has most likely undergone minute and subtle changes which escape our coarser means of examination—and the investigations of Dr. Sims shew that wasting of all or of parts of it is common. The senses have failed because the minute organization of their matériel has undergone certain modifications. Amidst this general mass of disorganizations it may be interesting and curious to single out one, and, making it the subject of special study, to see what share it has in destroying the rusty and worn-out machine—it may be interesting and curious as a matter of abstract pathological science, but in the eye of the utilitarian it will seem as nought, and the practical physician will be too apt to regard it as a pathological plaything—a subject for school-boys to pore over in the dead-house, or for doctors who see more of the dead than of the living to muse or to discourse upon in the closet or the lecture-room.

We do not participate in this practical contempt for abstract science. It is the mixture of abstraction with matter of fact, that gives to medicine much of its interest. The mere routinist who looks on all knowledge for just what it will bring, may put guineas in his pocket, but will seldom materially advance his art. The great improvements are rarely made by your mere practitioner. Genius and speculation discover truths, which men without genius or speculation apply. Mr. Pott was a much better practical surgeon than John Hunter, but how much more has John Hunter advanced the science of practical surgery than Mr. Pott. That one idea, that the ligature of an artery above an aneurism might cure an aneurysm, has done more for humanity, directly and indirectly, than the labours of almost all the empiric sect from Hippocrates to the meanest drug composer of this day. We say, then, to Dr. Sims—"Go on. Persevere in your laborious and scientific investigations."

### IV. Some Cases of Mental Derangement, successfully treated by the Acetate of Morphia. By Edward J. Seymour, M.D. &c.

This Paper is of a widely different character from that of the three we have quitted. After perusing pathological articles, we turn with pleasure to those which exhibit curable or cured disease. The mind finds repose and a gleam of satisfaction at the prospect which wooes it from the contemplation of death. A successful case is like a novel. The page closes with the cure, as with the marriage of the parties, and nothing but health and happiness are in the future.

The laudable object of Dr, Seymour is to shew the good effects that sometimes attend the exhibition of the acetate of morphia in cases of insanity. He observes with justice that too much has been attributed to inflammation of the brain in the production of mental derangement. Experience, he says, has shewn that blood-letting in maniacal cases, unconnected with disease of other organs than the brain, is seldom attended with good effect, and of late years the soothing system has very generally been adopted by medical men, and founded as it is on true principles of pathology, is more likely to lead to success than any other. To diminish the increased and morbidly acute perceptions, and to effect a decrease in the sensibility of the organ, and to reduce the exaggeration of its natural functions, seems to be the great object; and so far from such a condition being always the result of too great a flow of blood to the head, morbidly stimulating the organ, it often occurs in those who have rather less blood than usual for the maintenance of life in the brain, occasioned by excessive evacuations, watching, anxiety, and bad living.

The cases which form the subject of the present paper are three. Each is interesting.

*Case* 1. "A lady, aged about forty-eight, was attacked in the month of August, 1833, (after exposure to severe distress by the death of a relation who expired in her presence,) with mental derangement. Her usual habits of thinking were those of great deliberation; she had lived much in society, and from her station, mixed much with the world, nor had there at any time been any, even the slightest, indication of eccentricity or weakness of mind. Her mind was, at the time I saw her, filled with gloomy ideas; imaginary neglect of great and solemn duties, and a belief of having committed indescribable and even ill-defined crimes, constituted the principal features of her malady. Her bodily health was unusually robust, and she had scarcely ever suffered even from trifling bodily ailments. In the first instance the patient was bled, and took repeated doses of purgative medicine, but without any beneficial effect. The pulse was not weak; the nights were sleepless, and there was constant watchfulness present; there was no pain in the head, but a sense of weight was described, and there was restlessness of the body always present, so that the patient would often endeavour to jump out of bed and run about the room. During an unavoidable absence from London, the patient was seen by my friend Dr. Southey, and she was kept under nauseating doses of tartar emetic without any satisfactory result.

It was now resolved to try the morphia, and a grain of the acetate was ordered to be taken every night, the bowels to be kept open by small doses of castor oil; the severity of the symptoms became greatly diminished, and it occurred to me that the sedative effect of cold would greatly assist the operation of the remedy. Ice was therefore kept to the head in a bladder, day and night. The morphia never failed to procure a good night, and thus by degrees, without any other remedy, except those mentioned, the mind cleared up. The use of ice was gradually abandoned, but the morphia continued to be administered every night during three months, although all trace of insanity had disappeared six weeks from the commencement of the employment of the remedies, during ten days of which the ice was kept constantly to the head. No relapse whatever has occurred in this patient." 171.

*Case* 2. A married lady, æt. 34, the mother of several children, who had over-exerted herself previously to commencing a journey, and in whom the catamenia were reported to have been, in consequence, suddenly checked, was attacked with what was thought to be inflammation of the lungs. For this she was bled, &c., but the blood displayed no buffy coat. She was then seized with mania, the prevalent idea being fear of some object near her, or of some serious accident having happened to those she loved. Dr. Seymour saw her once and ordered a dose of morphia, which gave her sleep but was not repeated. In about a fortnight, the patient was removed to London, and placed under Dr. Seymour's cure. The mind was seriously affected, the patient was violent, and her imagination strangely disordered. She fancied her children were murdered, and their spirits returned to torment her, and that some similar evil menaced her husband. Her sleep was broken, and her conversation incoherent. The pulse was 100, and not strong. The tongue white, but not loaded; there was no fever, but occasional flushing of the face. The catamenia had returned at the usual period. A grain of the acetate of morphia was ordered to be given every night at bed time—ice was applied to the head—and the bowels were kept open by castor oil or senna every other morning. In three weeks the patient began to recover, but she was not well before several months had elapsed. On one occasion opium was substituted for the morphia, but it greatly disagreed.

*Case* 3. A lady whilst greatly distressed in mind bore a child. Her confinement was followed by mental derangement, and a state of utter imbecility. Aroused occasionally by the importunities of those who watched her, she uttered a short quick cry and relapsed into her usual state. Her fæces and urine were passed all unconsciously. The skin was cold, the pulse feeble, she never seemed to feel pain, nor did her hand wander to her head as if any painful affection of the brain existed. Morphia every night and castor oil every other morning were prescribed. She very slowly recovered and has since continued well.

Dr. Seymour adds—

"I will not detain the Society by more examples of the utility of this medicine in diseases of the mind, although I have seen several of a more chronic form experience great relief from its employment.

It appears to have been of more use in what is termed melancholia, where the mind is tortured by imaginary want, ruin, or crime, than where the derangement partakes of more brilliant ideas, as of super-human knowledge, dexterity, or wisdom; or in those cases in which one single idea occupies and absorbs the mental powers." 175.

The Paper is worthy of the consideration of our readers.

